# Trends, decomposition, and projections of gout burden among middle-aged adults in China: GBD 2023

**DOI:** 10.1097/MD.0000000000051086

**Published:** 2026-10-09

**Authors:** Chenyang Huang, Quanwei He, Hongwen Song, Zhuhui Duan, Zhiyu Wang, Sensen Cheng, Yueli Qi, Wei Xu, Huan Nie, Qianqing Guo, Xiaoying Sun, Yaqiong Fan, Zhiyan Mai, Haishuo He, Zhaowei Zhou, Wei Zhu

**Affiliations:** a Department of Nephrology and Rheumatology, The 989th Hospital of the PLA Joint Logistics Support Force, Luoyang, Henan Province, China; b Liver Disease Research Center, Hainan Hospital of Chinese PLA General Hospital, Sanya, Hainan Province, China; c Department of Dermatology, The 989th Hospital of the PLA Joint Logistics Support Force, Luoyang, Henan Province, China; d Department of Stomatology, Luoyang Central Hospital Affiliated to Zhengzhou University, Luoyang, Henan Province, China; e Department of Geriatrics, Shanghai Xuhui Central Hospital, Zhongshan-Xuhui Hospital, Fudan University, Shanghai, China.

**Keywords:** body-mass index, China, Global Burden of Disease, gout, kidney dysfunction

## Abstract

Gout has become a rapidly expanding metabolic-inflammatory disease in China, but the long-term trajectory, changing attributable risks, and future burden among middle-aged adults remain incompletely defined. We assessed gout burden among Chinese adults aged 40 to 59 years from 1990 to 2023 and projected trends to 2035. Using Global Burden of Disease Study 2023 (GBD 2023) data, we analyzed gout prevalence, incidence, and disability-adjusted life years among Chinese adults aged 40 to 59 years. Joinpoint regression identified temporal inflection points; Das Gupta decomposition quantified contributions from population growth, aging, and epidemiological change; Bayesian age-period-cohort modeling projected burden to 2035; and population attributable fractions (PAFs) were estimated for high body mass index (BMI) and kidney dysfunction, with sex- and age-stratified analyses. Prevalent cases increased from 2.56 million in 1990 to 6.75 million in 2023, while the age-standardized prevalence rate rose by 21.08% (1226.32–1484.81 per 1,00,000). The steepest acceleration occurred during 2009 to 2018. Population growth accounted for approximately 70% to 73% of the absolute increase, epidemiological change for 19% to 24%, and aging for 6% to 8%. High BMI remained the dominant attributable risk factor, accounting for 32.2% of gout-related disability-adjusted life years in 2023, whereas the PAF attributable to kidney dysfunction declined from 6.5% to 4.7%. Men had a higher BMI-attributable PAF at ages 40 to 44 years, but women had a greater proportional burden from age 45 onward. Projections indicated that gout burden would remain at a high level through 2035. Gout burden among middle-aged adults in China has risen substantially and is increasingly shaped by metabolic risk. Population-level weight and dietary interventions, sex-sensitive prevention and screening, and guideline-concordant urate-lowering management are needed to curb the projected burden. As an ecological analysis of modeled estimates, these findings identify population-level associations rather than establish causation.

## 1. Introduction

Gout is a chronic inflammatory arthritis caused by the deposition of monosodium urate crystals in joints and surrounding tissues, resulting in acute arthritis, tophi formation, severe joint pain, inflammation, and swelling.^[1]^ Beyond joint damage, gout is strongly associated with all-cause mortality, cardiovascular disease, chronic kidney disease, urolithiasis, and diabetes, with most of these associations being bidirectional and persistent.^[2]^ Hyperuricemia, the physiological prerequisite for gout, is similarly linked to hypertension, cardiovascular disease, diabetes, and metabolic syndrome, imposing a substantial public health burden on society.^[3,4]^ From a global epidemiological perspective, previously published GBD-based analyses reported that between 1990 and 2021 the global incidence of gout rose from 93.10 to 109.08 per 1,00,000, with incident cases increasing from approximately 3.98 to 9.40 million, and that prevalent cases rose from 9.14 to 19.24 million over the same period,^[5,6]^ indicating that gout has become one of the fastest-growing chronic inflammatory joint diseases worldwide.

The burden of gout in China has risen even more steeply over the past 3 decades, with growth rates substantially exceeding the global average. GBD 2021-based estimates indicate that between 1990 and 2021 the incidence of gout in China increased from 122.52 to 151.61 per 1,00,000, with incident cases rising from approximately 1.18 to 3.08 million, while prevalence increased from 640.68 to 810.36 per 1,00,000 and prevalent cases rose from approximately 5.97 to 16.79 million. With improvements in living standards, gout has emerged as another common metabolic disease in China following diabetes.^[6]^ Data from the China Kadoorie Biobank, a large prospective cohort study of 5,12,724 adults, further confirm that the incidence of gout among Chinese adults is 23.4 per 1,00,000 person-years and is significantly higher among men and older individuals; moreover, patients with gout exhibit markedly elevated risks of all-cause mortality (HR = 1.58), cardiovascular disease (HR = 1.87), chronic kidney disease (HR = 5.61), urolithiasis (HR = 2.50), and diabetes (HR = 1.99),^[3]^ underscoring that gout in China is no longer merely a “joint disease” but rather a multi-system metabolic-inflammatory syndrome.

Within the population distribution of gout in China, middle-aged adults aged 40 to 59 years represent a critical window of disease burden, for 4 key reasons. First, from an epidemiological perspective, the risk of developing gout increases markedly with age, and middle-aged and older populations – particularly men – are consistently identified as the highest-risk groups; in China the burden within the middle-aged range peaks in the 55 to 59 age group.^[7,8]^ Second, regarding disability burden, GBD-based analyses indicate that gout-related YLDs increased substantially between 1990 and 2021, with the highest counts concentrated in the mid-fifties to mid-sixties, suggesting that middle age is the period in which gout-related disability accumulates most intensively.^[8]^ Third, from a clinical trajectory perspective, although gout has traditionally been regarded as a disease of older adults, contemporary Chinese cohort data show that the age at onset has continued to shift younger. A recent multicentre cross-sectional cohort study of 10,658 Chinese patients with gout diagnosed between 2003 and 2022 found that the mean age at onset fell from 42.0 years in 2003 to 38.9 years in 2022 (*P* for trend < .001), with the proportion of patients whose disease began at ≤19 years and at 20 to 29 years increasing by 5.8% and 9.6% annually, respectively; patients in the second decade also had a higher burden of tophi, metabolic comorbidities, and adverse lifestyle exposures.^[9]^ These findings are consistent with an earlier single-center series of 9754 Chinese patients, in which the mean onset age was 4.14 years younger in 2013 to 2018 than in 2008 to 2012 and obesity was an independent predictor of early-onset disease.^[10]^ Taken together, these data imply that a large cohort of early-onset patients will enter the 40 to 59 age bracket over the next 10 to 20 years, forming an “early-onset to mid-life accumulation” trajectory and rendering this age group a critical clinical decision window for tophus formation, comorbidity development, and functional loss. Fourth, from a policy perspective, adults aged 40 to 59 years constitute a core target population for chronic disease prevention and control under China’s Healthy China 2030 initiative,^[11]^ making precise characterization of this group relevant to national chronic disease management strategies. We emphasize that the 40 to 59-year window was specified a priori on the basis of these 4 considerations, before any analysis of the GBD 2023 estimates was undertaken; it was not derived from data exploration, and no alternative age windows were tested and discarded.

Although several GBD-based studies have examined the burden of gout in China, systematic GBD analyses specifically targeting Chinese adults aged 40 to 59 years remain absent. Existing studies have separately focused on all-age populations (using GBD data with Joinpoint and ARIMA models to analyze gout trends in China and globally),^[8]^ young adults (showing that gout burden among Chinese adults aged 15 to 39 years is substantially higher than the global average),^[12]^ or older adults, leaving the 40 to 59 age range as a comparatively underexamined stratum. As early as the GBD 2017–based analysis of gout in China, the authors explicitly called for greater attention to body weight management in order to control the rising prevalence of gout in young and middle-aged people,^[5]^ yet to date no dedicated analysis of this group has been published. Furthermore, existing GBD studies of gout in China exhibit notable methodological limitations: most have relied solely on Joinpoint regression combined with ARIMA-based forecasting,^[8]^ without Das Gupta decomposition (separating the contributions of population growth, population aging, and epidemiological change), formal risk-factor attribution, or the more advanced Bayesian age-period-cohort (BAPC) framework for long-term projection. These gaps limit the ability to characterize the drivers and future trajectory of the disease burden. To address them, the present study leverages the most recent GBD 2023 database to systematically analyze the burden of gout among Chinese adults aged 40 to 59 years from 1990 to 2023 and to project trends through 2035. By applying an analytical framework that integrates Joinpoint regression, Das Gupta decomposition, BAPC-based forecasting, and risk factor attribution, this study aims to provide evidence to inform precision prevention strategies, optimize healthcare resource allocation, and support the implementation of China’s chronic disease management goals under Healthy China 2030. We deliberately restrict the analytical window to 40 to 59 years in order to characterize this stratum in depth; the consequences of excluding adults aged 60 years and older, in whom the absolute gout burden is also substantial and in whom kidney dysfunction is a more prominent attributable risk, are considered in the Limitations section.

## 2. Materials and methods

### 2.1. Data source and study population

Epidemiological estimates of gout burden were retrieved from the Global Burden of Diseases, Injuries, and Risk Factors Study 2023 (GBD 2023), accessed through the Global Health Data Exchange (GHDx) results tool maintained by the Institute for Health Metrics and Evaluation (IHME) at the University of Washington (https://vizhub.healthdata.org/gbd-results/). All methods described below follow the GBD 2023 specifications as reported in the GBD 2023 capstone publications, and GBD 2023 reestimates the entire 1990 to 2023 time series, superseding all previous GBD rounds.13, 14 Within the GBD 2023 framework, prevalence and incidence for most nonfatal conditions are modeled using Disease Modeling Meta-Regression (DisMod-MR; version 2.1; Institute for Health Metrics and Evaluation, University of Washington, Seattle, Washington, USA), a Bayesian meta-regression tool that enforces internal consistency between prevalence, incidence, remission, and mortality, while spatiotemporal Gaussian process regression or custom models are applied when Disease Modelling Meta-Regression cannot adequately capture the parameter distributions; meta-regression–Bayesian, regularized, trimmed is used to adjust for bias in input data and to perform age and sex splitting. Uncertainty is propagated throughout the estimation process. Mean estimates are based on 250 draws from the estimate distribution, with 95% uncertainty intervals (UIs) defined by the 2.5th and 97.5th percentiles of the draw distribution.13 This study is a secondary ecological analysis of publicly available, population-aggregated GBD 2023 estimates, and examined aggregated gout burden without direct involvement of human participants.

The analytic population was confined to Chinese residents aged 40 to 59 years, further partitioned into 4 contiguous 5-year strata (40–44, 45–49, 50–54, and 55–59 years). Sex-disaggregated estimates (male, female, and both sexes combined) were extracted annually from 1990 to 2023. Because all data were publicly accessible, population-aggregated, and fully de-identified, ethical approval and individual informed consent were not required.

### 2.2. Case definition and outcome indicators

Within the GBD nosological framework, gout is identified using ICD-10 codes M10–M10.9 and ICD-9 codes 274–274.9. GBD harmonizes data from heterogeneous sources under a standard gout case definition; the aggregated estimates used here do not distinguish crystal-proven from clinically diagnosed gout across data sources.

Four core burden indicators were examined: incidence, prevalence, years lived with disability (YLDs), and disability-adjusted life years (DALYs). In GBD 2023, YLDs are obtained by multiplying prevalence estimates by the severity-specific disability weight for each sequela, years of life lost are calculated as the product of the number of deaths and the GBD standard life expectancy at the age of death, and DALYs are the sum of years of life lost and YLDs.^[13,14]^ Because the GBD framework assigns no direct mortality to gout, DALYs for gout are composed entirely of YLDs and the 2 metrics are numerically identical. For each of the 4 indicators, both absolute case counts and age-standardized rates (ASRs) per 1,00,000 population were extracted for 1990 and 2023, with age standardization referenced to the GBD world standard population to facilitate cross-temporal and cross-stratum comparability. All point estimates are accompanied by 95% UIs.

### 2.3. Statistical analysis

All analyses were carried out in R software (version 4.4.1; R Foundation for Statistical Computing, Vienna, Austria), supplemented by the R interface to the Joinpoint Regression Program where applicable. Visualizations were produced with the R packages ggplot2, ggsci, patchwork, and maptools. Two-sided *P*-values below .05 were considered statistically significant; for Bayesian outputs, inference relied on whether the 95% credible interval (CrI) excluded the null.

#### 2.3.1. Temporal trend analysis: joinpoint regression and EAPC

To delineate inflection points in the temporal evolution of gout burden between 1990 and 2023, Joinpoint regression was performed via the R package segmented together with the National Cancer Institute Joinpoint methodology. A joinpoint regression model was applied to describe continuous changes using the grid-search method to fit the regression function with unknown joinpoints, and the number of significant joinpoints was identified through permutation tests in which each *P*-value was obtained using Monte Carlo methods, and the overall asymptotic significance level was preserved through a Bonferron correction.^[15]^ The natural logarithm of each ASR was modeled as a piecewise linear function of calendar year, and segment-specific annual percent changes (APCs) together with the average annual percent change (AAPC) across the entire interval were estimated, with statistical significance declared when the 2-sided *P*-value of the slope coefficient was below .05. In parallel, the estimated annual percentage change (EAPC) was derived from a log-linear regression of the ASR on calendar year, with the EAPC calculated as 100 × (exp(β) − 1) and its 95% confidence interval (CI) obtained from the standard error of β.

#### 2.3.2. Das Gupta decomposition analysis

To quantify the contribution of distinct demographic and epidemiological forces to the observed change in gout burden between 1990 and 2023, we applied the Das Gupta decomposition method as refined by Chevan and Sutherland.^[16]^ Under this framework, the total number of cases (or DALYs) in a given year is expressed as the product of 3 components: total population size, the age structure of that population (the proportion of the population in each 5-year stratum), and the age-specific rate of the outcome. The difference in the outcome between the 2 endpoint years is then partitioned additively into the effect attributable to population growth (change in total population size, holding age structure and age-specific rates at their standardized values), population aging (change in age structure, holding population size and age-specific rates constant), and epidemiological change (change in age-specific rates, holding population size and age structure constant). Because the decomposition is order-dependent when computed pairwise, effects were symmetrically standardized across all possible orderings of the factors so that the 3 components sum exactly to the observed total change. The decomposition was performed separately for incidence, prevalence, and DALYs, and separately for men, women, and both sexes combined. Results are reported both as absolute contributions (numbers of cases or person-years) and as percentage-point contributions to the total relative change.

#### 2.3.3. Bayesian age-period-cohort (BAPC) projection

Future burden trajectories from 2024 through 2035 were projected using a BAPC framework with integrated nested Laplace approximations, implemented via the R packages BAPC and integrated nested Laplace approximation.^[17]^ Second-order random-walk priors were specified on the age, period, and cohort effects to ensure smoothness, and the model was fitted to the observed counts under a Poisson likelihood with an offset for the corresponding population. Compared with conventional ARIMA forecasting, BAPC simultaneously accounts for nonlinear cohort dynamics, propagates parameter uncertainty into the predictive distribution, and has demonstrated favorable calibration in long-horizon projection of chronic disease burden.^[17]^ United Nations World Population Prospects 2024 medium-variant projections were adopted as the denominator for forecasting age-specific rates. Point estimates and 95% CrIs were obtained from the posterior predictive draws. Because model-fitted values for the observed period are generated jointly with the projections, fitted counts for 1990 to 2023 may differ marginally from the corresponding GBD 2023 input estimates reported in Section 3.1; both are reported to 3 or more significant figures and all such differences fall well within the associated UIs.

#### 2.3.4. Risk factor attribution analysis

Risk-factor-attributable burden was assessed using the GBD 2023 comparative risk assessment framework. For each risk–outcome pair supported by sufficient epidemiological evidence under the World Cancer Research Fund causal grading criteria, the population attributable fraction (PAF) was computed from the joint distribution of exposure in the population, the relative risk function relating exposure to the outcome, and the theoretical minimum risk exposure level, and was then applied to total gout burden to obtain the attributable burden.^[13]^ For gout, the GBD 2023 hierarchy identifies high body-mass index (BMI) and kidney dysfunction as the principal attributable risk factors; kidney dysfunction is defined by an estimated glomerular filtration rate below 60 mL/min/1.73 m² or an albumin-to-creatinine ratio of 30 mg/g or higher. Attributable DALYs and YLDs, attributable rates per 1,00,000 population, and the percentage contribution of each risk factor were extracted for 1990 and 2023, stratified by sex (male, female) and by 5-year age stratum (40–44 to 55–59 years). Absolute and percentage changes from 1990 to 2023 were computed for all estimates, and temporal trends were summarized using EAPC as described in Section 2.3.1. Because both PAFs and total burden are modeled quantities, attributable estimates are interpreted as population-level associations under the GBD causal framework rather than as individually estimated causal effects.

### 2.4. Reporting standard

Reporting of the present analysis adheres to the Guidelines for Accurate and Transparent Health Estimates Reporting statement.^[18]^

## 3. Results

### 3.1. Temporal trends in the burden of gout in China, 1990–2023

In China, the absolute burden of gout among middle-aged adults expanded markedly over the past 3 decades. Between 1990 and 2023, the number of prevalent cases increased from approximately 2.56 to 6.75 million (+163.69%), and incident cases rose from 0.49 to 1.26 million per year (+158.34%); the corresponding DALYs more than doubled, increasing from 82,061 to 2,16,025 person-years (+163.25%). When standardized to the world reference population, however, the magnitude of increase was substantially smaller: the age-standardized prevalence rate (ASPR) rose by 21.08% (from 1226.32 to 1484.81 per 1,00,000), the incidence rate by 19.74%, and the DALY rate by 21.35%. This contrast between the steep rise in absolute counts and the more modest increase in ASRs indicates that demographic changes contributed substantially to the expanding burden, while changes in age-specific disease rates were comparatively modest. The burden was consistently higher among males than females across all indicators, although the proportional increase in absolute case numbers was somewhat larger in females than in males (e.g., +179.81% vs +159.58% for prevalence; Table 1).

**Table 1:** Gout burden among middle-aged adults (aged 40–59 years) in China in 1990 and 2023, by sex.

Indicator	Sex	1990		2023		Relative change, 1990–2023 (%)	
		Number (95% UI)	ASR per 1,00,000 (95% UI)	Number (95% UI)	ASR per 1,00,000 (95% UI)	Number	ASR
Prevalence	Male	20,39,664 (13,20,026–29,26,663)	18,56.22 (1201.77–2661.28)	52,94,481 (34,53,578–76,39,082)	23,13.76 (1501.99–3349.17)	+159.58	+24.65
	Female	5,19,140 (3,28,555–7,59,206)	526.04 (333.06–767.87)	14,52,622 (9,29,957–20,93,267)	634.15 (403.88–920.08)	+179.81	+20.55
	Both sexes	25,58,804 (16,46,625–36,83,299)	12,26.32 (789.42–1763.61)	67,47,103 (43,92,251–97,19,358)	1484.81 (961.70–2148.80)	+163.69	+21.08
Incidence	Male	3,83,407 (2,26,419–5,81,006)	3,48.50 (205.44–528.38)	9,76,958 (5,66,810–14,75,456)	430.38 (251.57–651.27)	+154.81	+23.50
	Female	1,03,432 (60,015–1,57,778)	1,04.72 (60.67–159.86)	2,80,757 (1,61,974–4,24,778)	123.66 (71.92–187.65)	+171.44	+18.09
	Both sexes	4,86,839 (2,85,692–7,36,961)	2,33.05 (136.51–352.93)	12,57,715 (7,27,045–18,94,811)	279.06 (162.59–421.08)	+158.34	+19.74
DALYs/YLDs*	Male	65,357 (36,065–1,05,135)	59.45 (32.81–95.55)	1,69,632 (97,587–2,71,285)	74.34 (42.31–118.64)	+159.55	+25.03
	Female	16,704 (9018–27,233)	16.92 (9.15–27.58)	46,393 (25,615–74,574)	20.37 (11.13–32.69)	+177.74	+20.37
	Both sexes	82,061 (45,677–1,32,098)	39.31 (21.87–63.24)	2,16,025 (1,24,412–3,45,952)	47.70 (27.17–76.25)	+163.25	+21.35

Estimates were obtained from the Global Burden of Disease Study 2023. The number of cases (or DALYs/YLDs) is reported for the Chinese population aged 40 to 59 years, whereas rates are age-standardized to the GBD world standard population to allow comparability across years and sexes. Ninety-five-percent uncertainty intervals were derived from 250 draws and represent the 2.5th to 97.5th percentile range. Relative change was calculated as (Value2023 − Value1990)/Value1990 × 100%.

ASR = age-standardized rate, DALY = disability-adjusted life years, GBD = Global Burden of Disease, UI = uncertainty interval, YLD = years lived with disability.

* Under the GBD framework, gout is modeled as a nonfatal condition; consequently, DALYs comprise YLDs only and the 2 indicators yield identical estimates.

Across the 1990 to 2023 period, all 3 age-standardized metrics showed an overall upward trajectory (Fig. S1, Supplemental Digital Content 1). The ASPR increased from 1226.32 to 1484.81 per 100,000, while the age-standardized incidence and DALY rates followed broadly parallel patterns. The increases were more pronounced in males, among whom the age-standardized prevalence, incidence, and DALY rates rose by 24.65%, 23.50%, and 25.03%, respectively, compared with corresponding increases of 20.55%, 18.09%, and 20.37% in females (Table 1). Consequently, a substantial sex disparity persisted throughout the study period. The temporal patterns and changes in trend segments were further characterized using EAPC and Joinpoint regression analyses below.

### 3.2. Estimated annual percentage change (EAPC) of gout burden in China, 1990–2023

From 1990 to 2023, the EAPC values for all 4 indicators of gout burden in China – prevalence, incidence, DALYs, and YLDs – were positive, and the lower bounds of their 95% CIs all exceeded 0 (Table 2). Across every indicator, the EAPCs of the overall rates for adults aged 40 to 59 years were higher than those of the corresponding ASRs, and the EAPCs in males were higher than those in females.

**Table 2:** Estimated annual percentage change (EAPC) in gout burden in China, 1990–2023.

Measure	Sex	EAPC (95% CI), % per yr	
		Age-standardized rate	Overall rate (ages 40–59 yr)
Prevalence	Male	1.04 (0.94–1.14)	1.23 (1.10–1.36)
	Female	0.77 (0.66–0.87)	1.05 (0.89–1.20)
	Both sexes	0.91 (0.81–1.01)	1.12 (0.99–1.26)
Incidence	Male	1.00 (0.89–1.11)	1.16 (1.03–1.30)
	Female	0.75 (0.64–0.86)	1.00 (0.85–1.14)
	Both sexes	0.88 (0.77–0.98)	1.06 (0.92–1.20)
DALYs/YLDs*	Male	1.04 (0.94–1.13)	1.22 (1.09–1.34)
	Female	0.76 (0.66–0.86)	1.02 (0.88–1.17)
	Both sexes	0.90 (0.81–1.00)	1.11 (0.98–1.24)

EAPC values were derived from a generalized linear regression model fitted to the natural logarithm of the annual age-standardized rate or overall rate among adults aged 40 to 59 years, expressed as a percentage change per year. A trend was deemed statistically significant when the EAPC point estimate and the lower bound of its 95% CI were both greater than 0 (indicating an increase) or both less than 0 (indicating a decrease). All estimates were derived from the Global Burden of Disease Study 2023.

CI =confidence interval, DALY = disability-adjusted life year, EAPC = estimated annual percentage change, YLD = years lived with disability.

* Under the Global Burden of Disease (GBD) framework, gout is classified as a nonfatal condition; consequently, DALYs are entirely attributable to YLDs, and the corresponding EAPC estimates are identical for the 2 indicators.

The EAPC for the overall prevalence rate among adults aged 40 to 59 years was 1.12% per year (95% CI: 0.99–1.26) in the total population, 1.23% (95% CI: 1.10–1.36) in males, and 1.05% (95% CI: 0.89–1.20) in females. The corresponding EAPCs for the ASPR were 0.91% (95% CI: 0.81–1.01) overall, 1.04% (95% CI: 0.94–1.14) in males, and 0.77% (95% CI: 0.66–0.87) in females. The EAPC for the overall incidence rate among adults aged 40 to 59 years was 1.06% per year (95% CI: 0.92–1.20) in the total population, 1.16% (95% CI: 1.03–1.30) in males, and 1.00% (95% CI: 0.85–1.14) in females. The age-standardized incidence rate increased at an EAPC of 0.88% (95% CI: 0.77–0.98) overall, 1.00% (95% CI: 0.89–1.11) in males, and 0.75% (95% CI: 0.64–0.86) in females.

Because gout was modeled as a nonfatal condition under the GBD framework, DALYs were entirely composed of YLDs, and the 2 indicators returned identical EAPC estimates. The EAPC for the overall DALY/YLD rate among adults aged 40 to 59 years was 1.11% per year (95% CI: 0.98–1.24) in the total population, 1.22% (95% CI: 1.09–1.34) in males, and 1.02% (95% CI: 0.88–1.17) in females. The corresponding ASRs increased at an EAPC of 0.90% (95% CI: 0.81–1.00) overall, 1.04% (95% CI: 0.94–1.13) in males, and 0.76% (95% CI: 0.66–0.86) in females.

### 3.3. Joinpoint regression analysis of age-standardized rates of gout in China

To further characterize the long-term trajectory of gout burden among middle-aged adults in China from 1990 to 2023, Joinpoint regression was applied to the ASRs of DALYs, incidence, and prevalence (Fig. 1, Table 3). All 3 indicators exhibited a significant and sustained upward trend throughout the study period, with AAPCs of 0.61% (95% CI: 0.58–0.64) for DALYs, 0.56% (0.54–0.59) for incidence, and 0.61% (0.59–0.64) for prevalence (all *P* < .001). Apart from a brief decline in the early 1990s, all 3 measures entered a prolonged phase of accelerating growth lasting nearly 2 decades from the late 1990s onward, peaking around the 2010s: the most rapid increases were observed during 2009 to 2018 for DALYs (APC = 1.44%, *P* < .05), 2010–2015 for incidence (APC = 1.79%, *P* < .05), and 2010 to 2017 for prevalence (APC = 1.59%, *P* < .05), suggesting that this decade represented a critical window for the rapid accumulation of gout burden in China.

**Table 3:** Average annual percentage change (AAPC) of age-standardized DALYs, incidence, and prevalence rates of gout in China, 1990–2023.

Measure	Sex	AAPC (%)	95% CI	*P*-value
DALYs	Both	0.61	0.58–0.64	<.001
	Female	0.58	0.55–0.60	<.001
	Male	0.72	0.69–0.75	<.001
Incidence	Both	0.56	0.54–0.59	<.001
	Female	0.51	0.49–0.53	<.001
	Male	0.65	0.64–0.67	<.001
Prevalence	Both	0.61	0.59–0.64	<.001
	Female	0.58	0.56–0.60	<.001
	Male	0.71	0.69–0.74	<.001

AAPC = average annual percentage change, CI = confidence interval, DALY = disability-adjusted life years.

**Figure 1.**
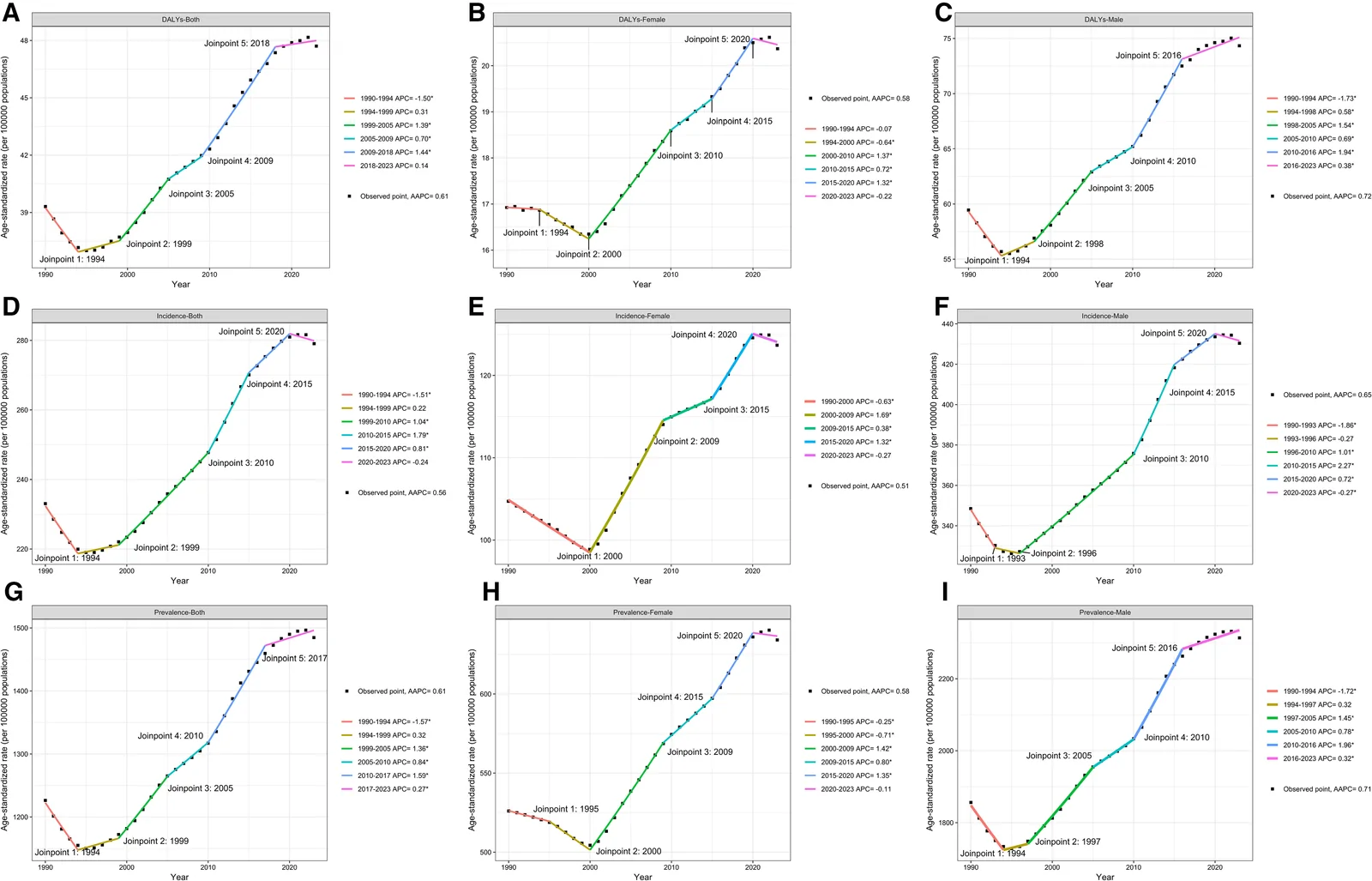
Joinpoint regression analysis of age-standardized rates of gout burden among middle-aged adults in China, 1990–2023. Panels (A–C) show DALYs, panels (D–F) incidence, and panels (G–I) prevalence for both sexes, females, and males, respectively. Observed annual rates and fitted Joinpoint segments are shown; segment-specific annual percentage changes (APCs) and overall average annual percentage changes (AAPCs) are displayed in each panel. APC =annual percentage change, AAPC = average annual percentage change.

Sex-stratified analyses further revealed pronounced male–female disparities, with males consistently exceeding females in terms of the timing, pace, and magnitude of the increase. The AAPCs in males reached 0.72% (DALYs), 0.65% (incidence), and 0.71% (prevalence), all higher than the corresponding values in females (0.58%, 0.51%, and 0.58%, respectively). Notably, the segment-specific APC peaked at 2.27% for male incidence during 2010 to 2015 and 1.96% for male prevalence during 2010 to 2016, representing the highest growth rates observed across all segments. Moreover, the accelerating phase began earlier in males (around 1996–1998) than in females, in whom the inflection point was generally delayed until around 2000, indicating an earlier exposure of men to gout-related risk factors. Although the growth rates moderated during 2020 to 2023, with several APCs approaching 0 or slightly negative, this short-term fluctuation may largely reflect disruptions in healthcare-seeking behavior and data ascertainment during the COVID-19 pandemic, while the overall burden of gout in China remained at a historically high level.

### 3.4. Decomposition analysis of changes in gout burden, 1990–2023

Using the Das Gupta decomposition method, we partitioned the changes in gout burden among middle-aged adults between 1990 and 2023 into 3 components: population growth, population aging, and epidemiological change (Figs. 2 and 3). Between 1990 and 2023, gout incidence, prevalence, and DALYs among middle-aged adults (both sexes combined) increased by 158.3%, 163.7%, and 163.3%, respectively. Among men, the corresponding increases were 154.8%, 159.6%, and 159.5%, whereas women experienced even larger relative increases of 171.4%, 179.8%, and 177.7%.

**Figure 2.**
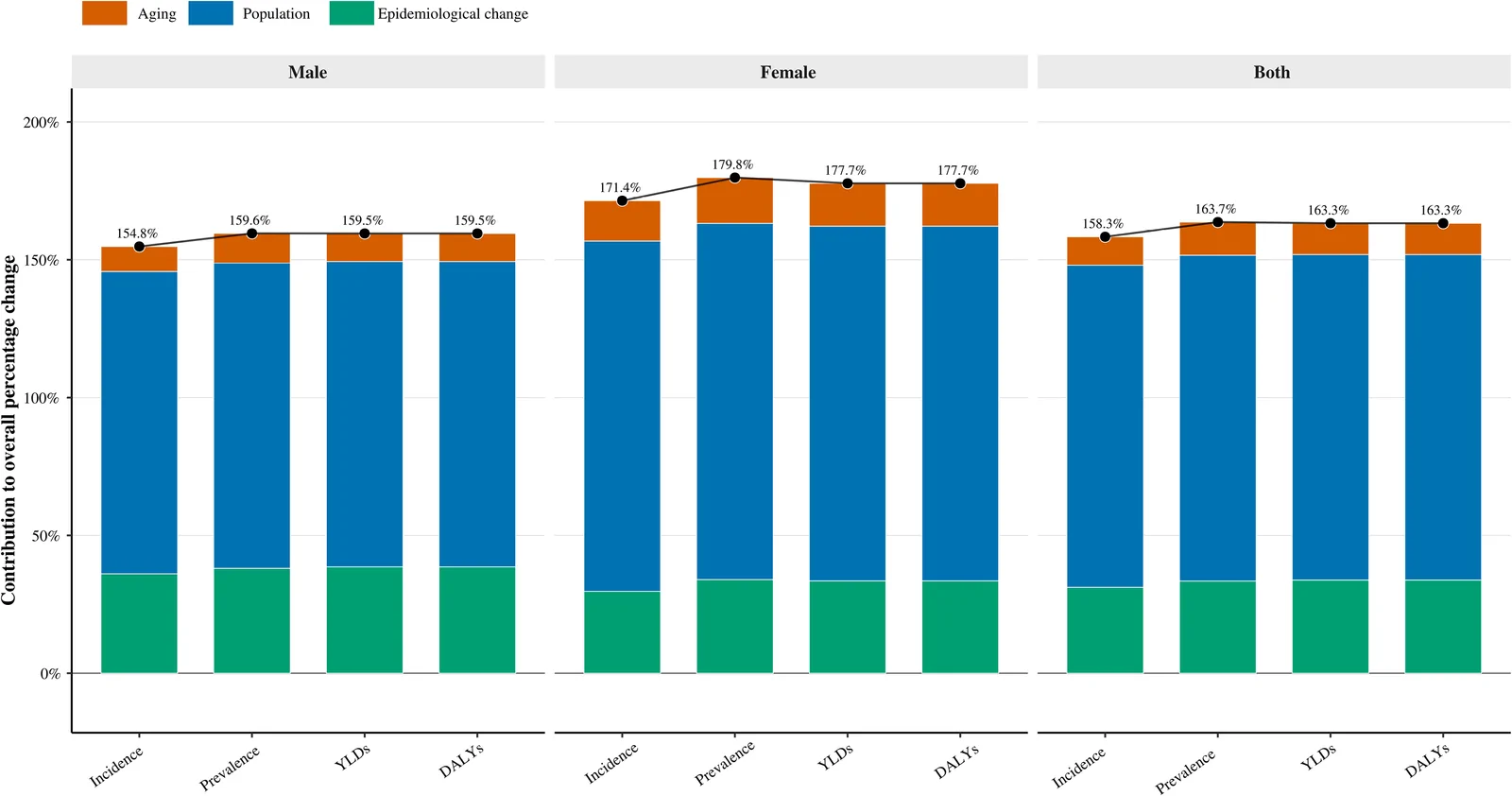
Decomposition of changes in gout burden among middle-aged adults in China, 1990–2023. Bars show the contributions of population aging, population growth, and epidemiological change to the overall percentage change; black dots indicate the overall percentage change between 1990 and 2023. DALY = disability-adjusted life-year, YLD = years lived with disability.

**Figure 3.**
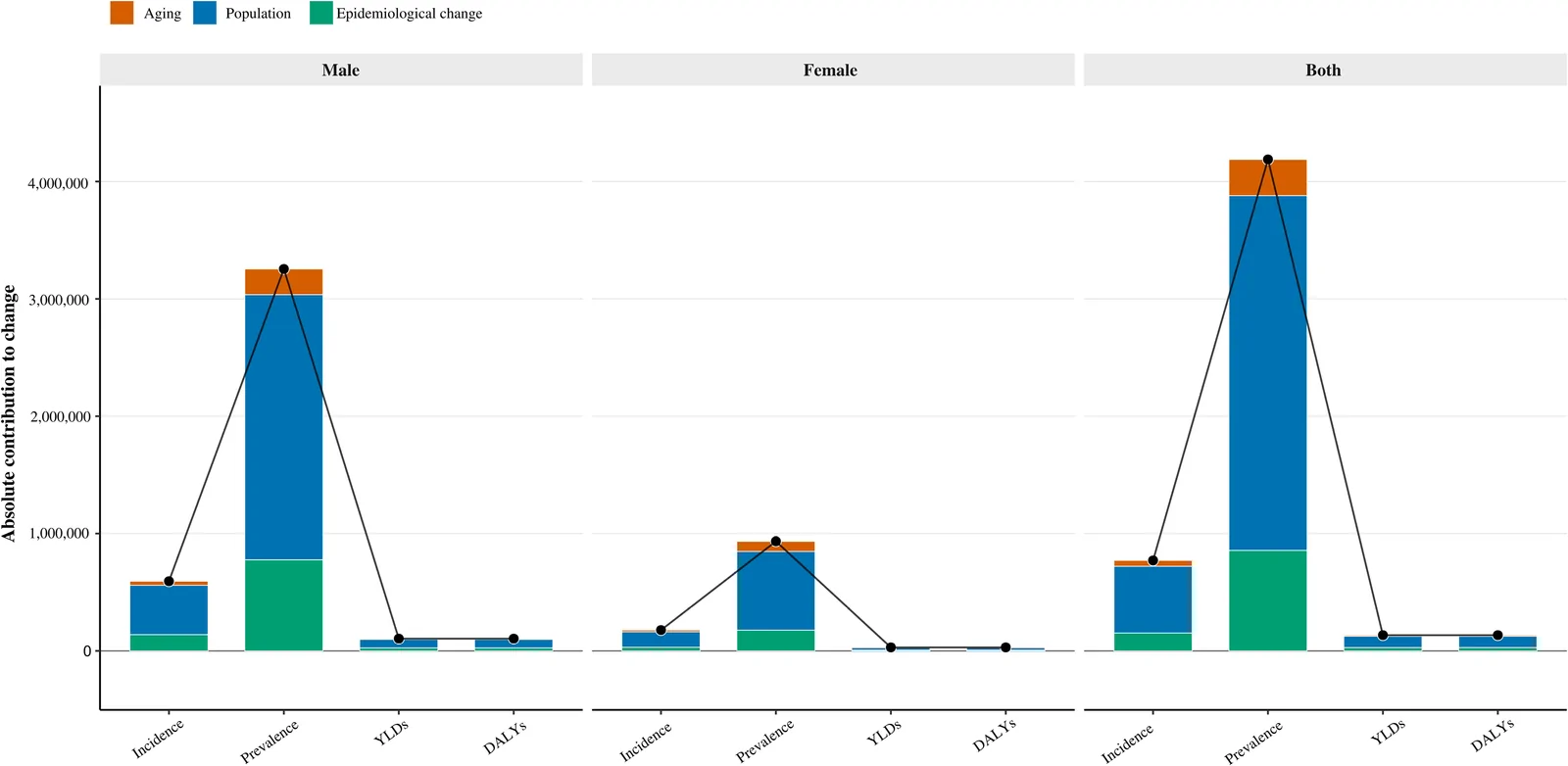
Absolute decomposition of changes in gout burden among middle-aged adults in China, 1990–2023. Bars show the absolute contributions of population aging, population growth, and epidemiological change; black dots indicate the overall absolute difference between 1990 and 2023. DALY = disability-adjusted life-year, YLD = years lived with disability.

Across all burden metrics (Fig. 2), population growth was the dominant driver, contributing approximately 110 percentage points (≈70% of the total change) for DALYs in men and 130 percentage points (≈73%) in women. Epidemiological change was the second largest contributor, accounting for ~38 pp (24%) in men and ~33 pp (19%) in women, indicating that age-specific rates of gout have themselves risen substantially. The contribution of population aging was comparatively modest (~10 pp [6%] in men; ~15 pp [8%] in women), reflecting the relatively narrow 20-year age window examined in this study.

In absolute terms (Fig. 3), gout prevalence increased by approximately 4.19 million cases between 1990 and 2023 (men: ∼3.25 million; women: ∼0.93 million) – the largest absolute change across all metrics. Incident cases increased by ∼ 7,71,000 per year (men: ∼5,94,000; women: ∼1,77,000). The pattern of absolute contributions mirrored that of the relative decomposition, with population growth dominating across all metrics. Notably, women exhibited larger relative increases than men across all 3 decomposition metrics (DALYs: 177.7% vs 159.5%), with slightly greater proportional contributions from both aging and population growth, suggesting that gout burden is rising more rapidly among middle-aged Chinese women.

### 3.5. BAPC projections of gout burden in China, 2024–2035

Using the BAPC model fitted to GBD 2023 data from 1990 to 2023, we projected the prevalence, incidence, and DALYs of gout among middle-aged adults in China through 2035 (Fig. 4). Detailed annual sex-specific estimates of incidence numbers and rates, DALY numbers and rates, and prevalence numbers and rates are provided in Tables S1–S2, S3–S4, and S5–S6, Supplemental Digital Content 2, respectively.

**Figure 4.**
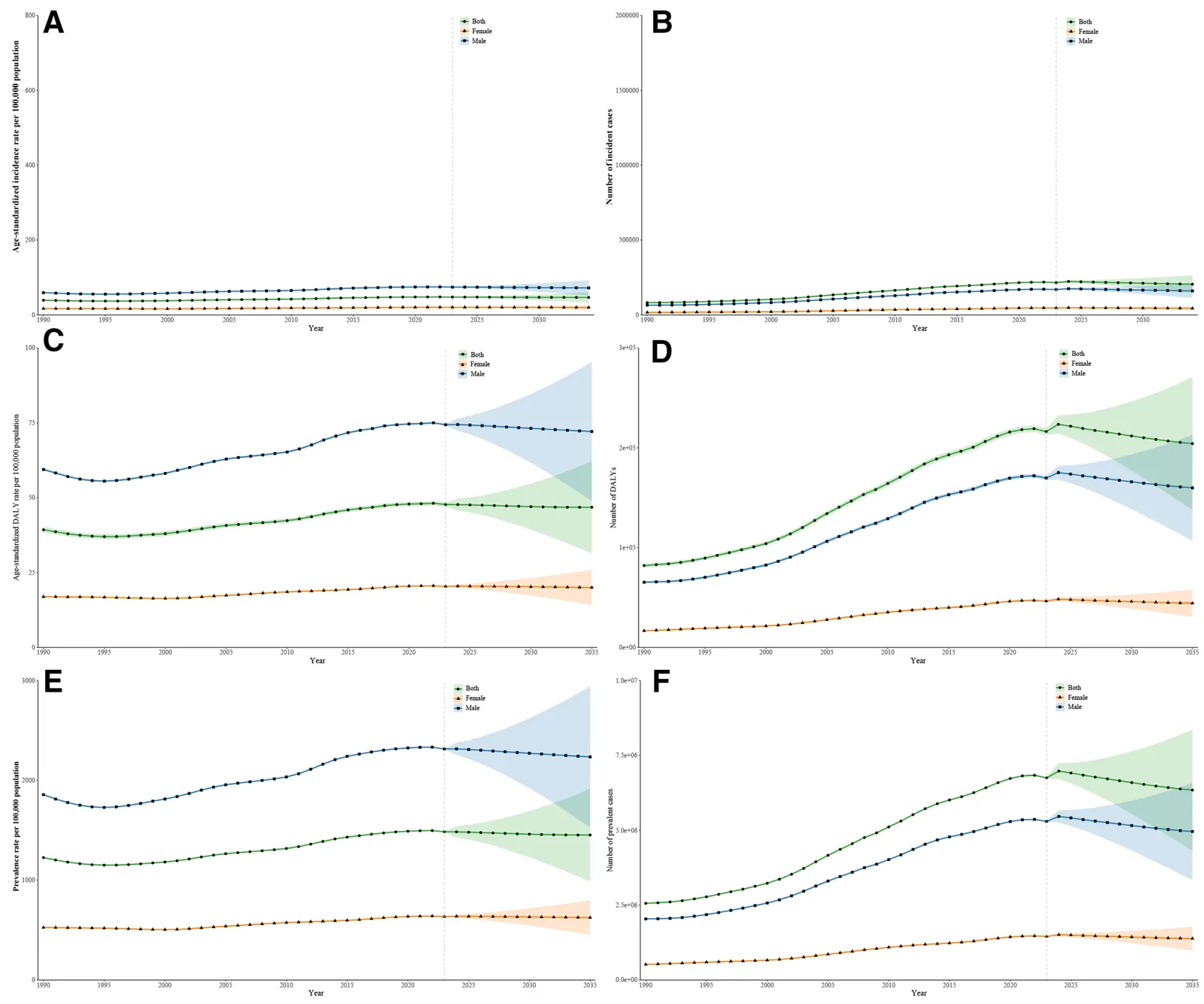
Bayesian age-period-cohort projections of gout burden among middle-aged adults in China, 1990–2035. Observed GBD 2023 estimates for 1990 to 2023 and BAPC projections for 2024 to 2035 are shown for prevalence, incidence, and DALYs, including numbers and rates, with sex-specific trajectories. Shaded intervals denote 95% credible intervals where displayed. BAPC = Bayesian age-period-cohort, DALY = disability-adjusted life-year, GBD = Global Burden of Disease.

Among the observed years, the number of prevalent gout cases increased from approximately 2.56 million (95% CrI: 2.55–2.57) in 1990 to 6.83 million (95% CrI: 6.81–6.85) in 2022, followed by a slight decline to 6.75 million in 2023. The BAPC projections indicated a transient increase to approximately 6.97 million cases in 2024, followed by a gradual decline to 6.34 million (95% CrI: 4.32–8.36) by 2035. The corresponding prevalence rate decreased gradually over the projection period, from 1483.64 per 1,00,000 in 2024 to 1452.44 (95% CrI: 986.22–1918.67) in 2035. A pronounced sex disparity was projected to persist: by 2035, the prevalence rate was estimated at 2234.43 per 1,00,000 in males compared with 625.17 per 1,00,000 in females, representing an approximately 3.6-fold difference (Tables S5 and S6, Supplemental Digital Content 6).

A similar pattern was observed for incidence. The annual number of new gout cases increased from 4,86,791 (95% CrI: 4,81,642–4,91,940) in 1990 to approximately 1.28 million in 2022 and 1.26 million in 2023. The projected number increased transiently to 1.30 million in 2024 and subsequently declined to 1.18 million (95% CrI: 0.79–1.57 million) by 2035. The corresponding incidence rate was projected to decline from 278.77 per 1,00,000 in 2024 to 271.14 (95% CrI: 181.76–360.53) in 2035. Sex-specific projections again showed a persistent male predominance, with incidence rates of 412.98 per 1,00,000 in males and 120.44 per 1,00,000 in females in 2035, a difference of approximately 3.4-fold (Tables S1 and S2, Supplemental Digital Content 2).

Gout-related DALYs increased from 82,036 person-years (95% CrI: 80,031–84,040) in 1990 to 2,19,098 (95% CrI: 2,15,771–2,22,424) in 2022, before declining slightly to 2,16,181 in 2023. The projected number of DALYs increased transiently to 2,23,508 in 2024 and then decreased gradually to 2,03,962 person-years (95% CrI: 1,37,376–2,70,549) by 2035. The corresponding DALY rate was projected to decline modestly from 47.70 per 1,00,000 in 2024 to 46.78 (95% CrI: 31.43–62.13) in 2035. The male predominance was expected to persist throughout the projection period, with the 2035 DALY rate remaining approximately 3.6 times higher in males than in females (72.14 vs 20.01 per 1,00,000; Tables S3 and S4, Supplemental Digital Content 4).

### 3.6. Decomposition of risk-attributable gout burden

According to the GBD 2023 framework, we examined the gout-attributable burden associated with the 2 risk factors quantified for gout – high body mass index (BMI) and kidney dysfunction – which showed divergent trajectories from 1990 to 2023 (Fig. 5). The number of high BMI-attributable DALYs increased nearly 4-fold, from approximately 17,874 person-years in 1990 to 68,914 person-years in 2023, whereas kidney dysfunction–attributable DALYs rose far more modestly, from 5493 to 11,990 person-years (Fig. 5A). Accordingly, the relative contribution of high BMI rose from 76.5% to 85.2%, while that of kidney dysfunction declined from 23.5% to 14.8% (Fig. 5B). In 2023, attributable DALY rates increased monotonically with age and were 3- to 4-fold higher in men than in women, peaking at 30.89 and 7.78 per 1,00,000 for high BMI and kidney dysfunction, respectively, in the male 55 to 59 group (Fig. 5C). Between 1990 and 2023, high BMI-attributable DALY rates rose substantially across all subgroups, with the largest increases in men aged 45 to 49 (+97.0%), 50 to 54 (+87.3%), and 55 to 59 (+86.3%), and pooled increases of 68.4% to 90.6% across sexes (Fig. 5D). In contrast, kidney dysfunction–attributable rates remained essentially stable in men (−3.3% to +0.8%) but declined markedly in women (−15.7% to −21.1%). These findings indicate an increasing predominance of BMI-related attributable burden relative to kidney dysfunction among the 2 risks quantified for gout in GBD 2023.

**Figure 5.**
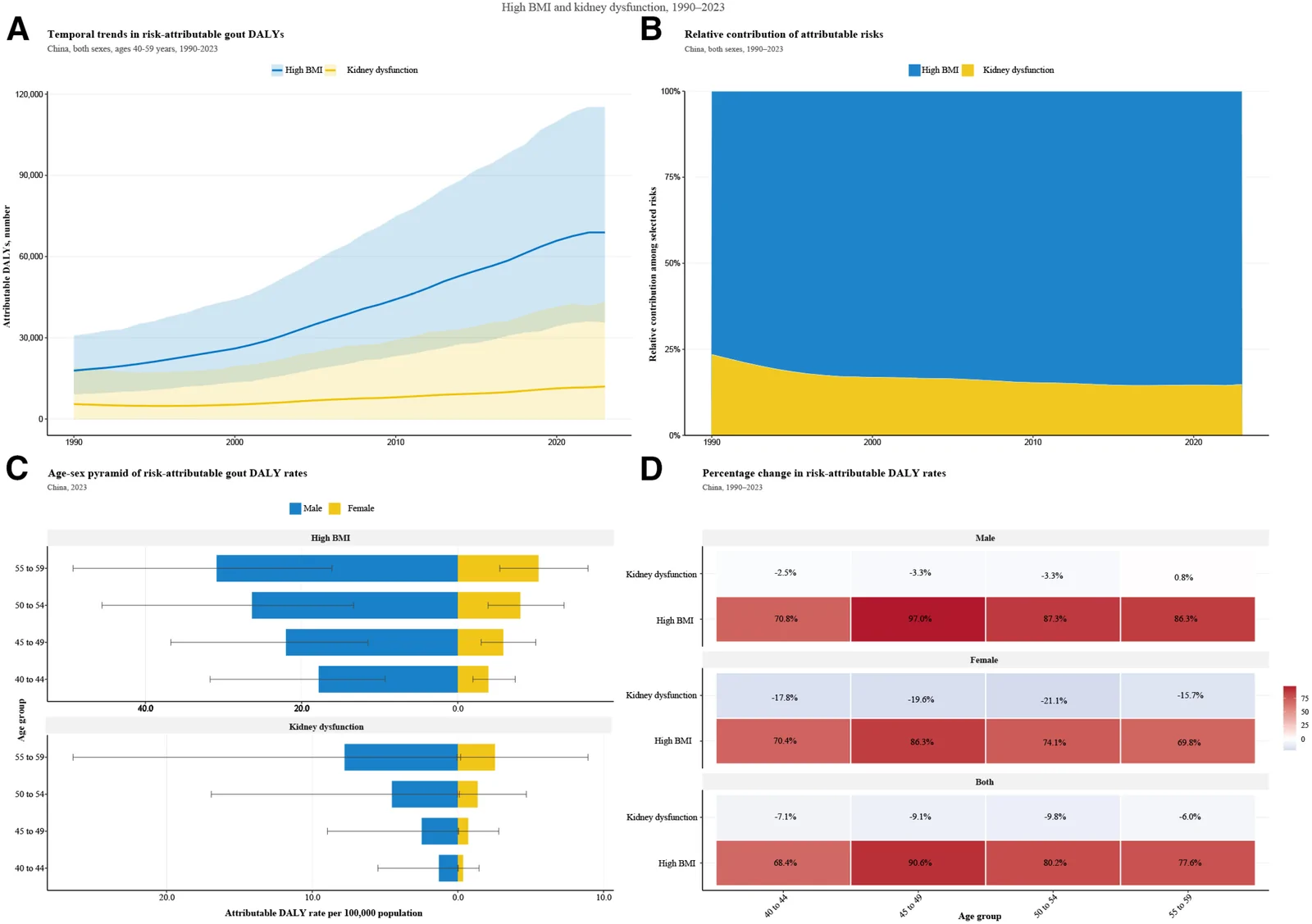
Risk-attributable burden of gout among middle-aged adults in China, 1990–2023. Panel (A) shows temporal trends in gout DALYs attributable to high body mass index (BMI) and kidney dysfunction; panel (B) shows their relative contributions; panel (C) shows age- and sex-specific attributable DALY rates in 2023; and panel (D) shows percentage changes in attributable DALY rates from 1990 to 2023. BMI = body mass index, DALY = disability-adjusted life-year.

### 3.7. Population-attributable fraction of gout DALYs

We estimated the PAF of gout DALYs for high BMI and kidney dysfunction among middle-aged adults from 1990 to 2023 (Fig. 6; Table S7, Supplemental Digital Content 8). High BMI remained the dominant attributable driver, with its pooled PAF rising from 21.9% in 1990 to 32.2% in 2023 – a relative increase of nearly 47% – whereas the PAF for kidney dysfunction declined modestly from 6.5% to 4.7% (Fig. 6A), indicating an increasing relative contribution of high BMI compared with kidney dysfunction. The increase in high-BMI-attributable burden was consistent across all age strata, with the 2023 PAF for both sexes combined ranging from 31.4% at 55 to 59 years to 33.5% at 40 to 44 years; the steepest relative acceleration occurred in the 45 to 49 group, in which the PAF rose from 20.4% to 32.1% (+11.7 percentage points, +57.4%). Kidney dysfunction–attributable PAF showed a clear age-increasing gradient, rising from 2.6% at 40 to 44 years to 8.0% at 55 to 59 years in 2023, together with mild attenuation over time (Fig. 6B). In 2023, sex-divergent patterns were observed for high BMI: men had a higher PAF than women at 40 to 44 years (34.1% vs 31.1%), but this male excess was already reversed by 45 to 49 years (32.0% in men vs 32.5% in women) and the female excess widened thereafter, with women bearing a greater attributable burden in the 50 to 54 (33.8% vs 31.0%) and 55 to 59 (33.8% vs 30.7%) strata (Fig. 6C). Between 1990 and 2023, all age strata exhibited substantial absolute gains in high-BMI-attributable PAF (+9.5 to +11.7 percentage points) alongside modest declines for kidney dysfunction (−0.7 to −2.5 percentage points; Fig. 6D). The reduction in kidney dysfunction–attributable PAF was consistently larger in women than in men across every age stratum (for example, −3.9 vs −2.2 percentage points at 55 to 59 years).

**Figure 6.**
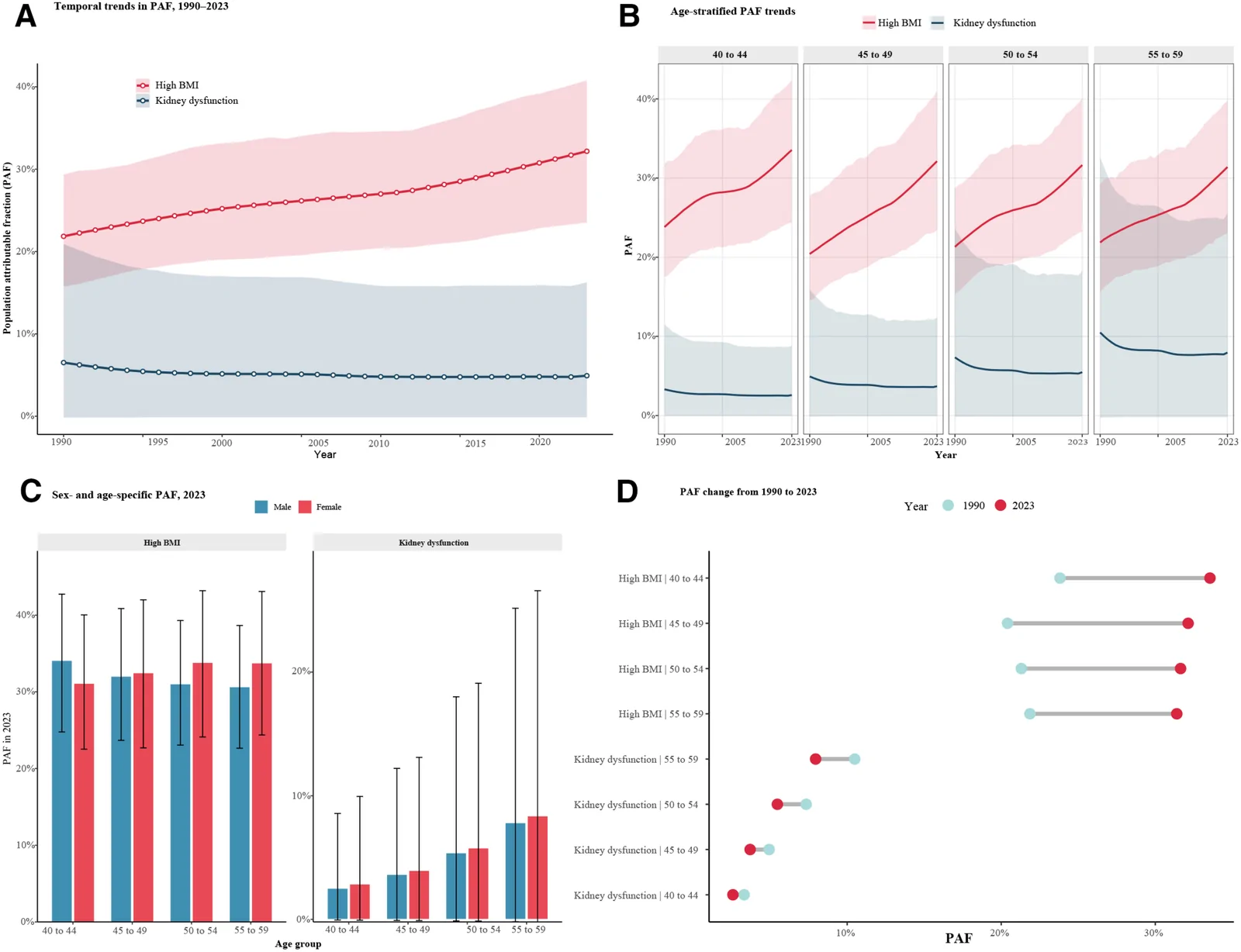
Population attributable fraction of gout DALYs among middle-aged Chinese adults, 1990–2023. Panel (A) shows temporal trends in population attributable fractions (PAFs); panel (B) shows age-stratified PAF trends; panel (C) shows sex- and age-specific PAFs in 2023; and panel (D) shows changes in PAFs from 1990 to 2023 for high BMI and kidney dysfunction. DALY = disability-adjusted life-year, PAF = population attributable fraction.

## 4. Discussion

This study presents the most comprehensive characterization to date of the gout burden among middle-aged adults in China across the 1990 to 2023 period, combining multiple analytical lenses to disentangle the demographic, epidemiological, and etiological forces that have reshaped this disease. The headline finding is clear: gout has transformed from a comparatively niche clinical concern into a chronic metabolic epidemic, with the absolute number of prevalent cases expanding from approximately 2.56 million in 1990 to 6.75 million in 2023, and the corresponding ASPR climbing by 21.08% despite the demographic compression imposed by China’s aging trajectory. What renders this trajectory particularly concerning is its persistence; Joinpoint analysis localized the steepest acceleration to the decade between 2009 and 2018, BAPC projections suggest that the burden will remain at a high level through 2035, and the attribution analyses point to a reconfiguration of the underlying risk landscape, in which high body-mass index has become increasingly dominant relative to kidney dysfunction among the 2 attributable risks quantified for gout in GBD 2023.

These observations align with – but substantially extend – the existing global and regional literature. Previous GBD-based analyses have shown that gout incidence rose from approximately 93 to 109 per 1,00,000 worldwide between 1990 and 2021, with absolute incident cases more than doubling, and East Asia exhibiting some of the steepest regional increases.^[8]^ National survey evidence from China has consistently confirmed that the burden is concentrated among working-age men, with the TIDE (Thyroid Disorders, Iodine Status and Diabetes: A National Epidemiological Survey) national survey reporting weighted prevalence rates of hyperuricemia and gout of 17.7% and 3.2%, respectively, alongside a more than 2-fold male predominance.^[19]^ Our restriction of the decomposition and attribution analyses to the 40 to 59-year window – a previously underexamined demographic stratum – adds a complementary perspective on what may be termed the middle-aged epidemic of gout, a phase of life during which metabolic risk accumulation, occupational stress, and dietary excess converge most intensively. The temporal inflection we identified in the late 1990s coincides closely with the broader nutritional and metabolic transition documented in the China Health and Nutrition Survey, in which adult obesity prevalence rose from approximately 4% in the early 1990s to roughly 16% by the mid-2010s in parallel with rapid urbanization, dietary Westernization, and declining occupational physical activity.^[20]^ The temporal coherence between these exposure trajectories and the gout incidence curve we observed is consistent with a genuine increase in underlying risk rather than a purely diagnostic-detection artifact, although ecological concordance of this kind cannot by itself establish causation.

A key finding of our analysis is the increasing dominance of high body-mass index among the 2 gout risk factors quantified in GBD 2023. The biological plausibility of the strong association between adiposity and gout is well established. Mendelian randomization studies have demonstrated that genetically higher BMI – but not waist-to-hip ratio adjusted for BMI – is causally associated with both gout risk and serum urate, with each standard-deviation increase in genetically predicted BMI conferring an odds ratio of approximately 2.24 for gout and a 0.30 mg/dL elevation in serum urate concentration.^[21]^ Subsequent multivariable Mendelian randomization work has refined this estimate by identifying insulin resistance and adipocytokine signaling as principal mediating pathways, while excluding meaningful reverse causation from urate to adiposity.^[22]^ The clinical magnitude of this gradient is striking: a dose-response meta-analysis of 10 prospective cohorts encompassing nearly 28,000 incident gout cases demonstrated that each 5 kg/m^2^ increase in BMI was associated with a 55% elevation in gout risk (summary RR 1.55, 95% CI: 1.44–1.66), and contemporary evidence syntheses continue to identify adiposity as the leading modifiable determinant of both hyperuricemia and incident gout.^[23,24]^ Mechanistically, adiposity raises serum urate through several interlocking pathways, including insulin-mediated reductions in renal urate excretion, increased hepatic purine turnover via the de novo purine biosynthesis pathway, leptin-driven proinflammatory signaling, and altered expression of urate transporters including ABCG2 and SLC2A9.^[25,26]^ Diet quality has also played a pivotal role in this transition: the Chinese dietary pattern has progressively Westernized over recent decades, driven principally by rising red-meat consumption, increasing intake of sugar-sweetened beverages, and declining whole-grain consumption.^[27]^ The purine and fructose load embedded in this dietary shift acts both directly – through increased exogenous substrate availability – and indirectly via weight gain to elevate serum urate, thereby converging on the hyperuricemic phenotype that underlies gout pathogenesis.^[24]^

The apparent attenuation of kidney dysfunction–attributable PAF – from 6.5% in 1990 to 4.7% in 2023 – warrants careful interpretation. This represents a relative rather than an absolute decline; the number of kidney dysfunction–attributable DALYs in fact more than doubled in absolute terms over the study period. The observed reduction in PAF therefore reflects primarily the disproportionate expansion of BMI-attributable burden, which mathematically compresses the proportional contribution of competing risk factors. Improvements in the detection and management of chronic kidney disease in China over the same period may have contributed additionally, although our data do not permit this to be tested directly. The persistent age-gradient we observed for kidney dysfunction–attributable PAF – rising monotonically from 2.6% at age 40 to 44 years to 8.0% at age 55 to 59 years in 2023 – is consistent with global evidence demonstrating that gout, primarily driven by hyperuricemia, retains a substantial component of renal etiology, with kidney dysfunction defined as an estimated glomerular filtration rate below 60 mL/min/1.73 m^2^ or an albumin-to-creatinine ratio of 30 mg/g or higher conferring a graded increase in risk.^[28]^ Importantly, the bidirectional nature of the urate–kidney axis means that hyperuricemia accelerates renal injury through endothelial dysfunction, oxidative stress, and tubulointerstitial inflammation, while renal impairment in turn reduces urate excretion.^[4]^ Kidney dysfunction should therefore be conceptualized not as a diminishing concern but rather as an enduring co-pathology that becomes increasingly relevant with advancing age and that is likely to reemerge as a dominant driver in older Chinese cohorts not captured within our 40 to 59-year analytical window.

A further noteworthy observation is the sex-divergent pattern in BMI-attributable PAF, with men exhibiting a higher attributable fraction than women at age 40 to 44 years, but the pattern reversing from age 45 onward, such that women bore a greater proportional burden in the 50 to 54 and 55 to 59 age strata. We hypothesize that this crossover is related, at least in part, to the perimenopausal decline in estrogen-mediated uricosuria. We emphasize, however, that our data contain no direct measure of menopausal status, and this interpretation is therefore a hypothesis generated by the analysis rather than a conclusion demonstrated by it. Two considerations bear on its plausibility. First, population-based evidence has established that menopause is independently associated with higher serum uric acid concentrations, whereas postmenopausal hormone use is associated with lower urate levels and reduced incident gout risk,^[29,30]^ with the principal mechanism thought to involve estrogen-dependent upregulation of renal urate transporters that promote excretion, an effect attenuated following ovarian senescence.^[26]^ Second, the timing is broadly compatible: in the China Kadoorie Biobank, the mean age at natural menopause among 87,349 Chinese postmenopausal women was 48.7 years (SD 4.3), with a median of 49.0 years,^[31]^ and the 45 to 49-year age stratum plausibly overlaps with the menopausal transition in a substantial proportion of women. The 45-year cutoff should nonetheless be regarded as an approximate proxy for the onset of the menopausal transition rather than as a marker of menopause itself, and the 5-year granularity of GBD age strata precludes any finer temporal alignment. Alternative, nonhormonal explanations must also be considered and cannot be excluded by our data. The most important is differential change in adiposity itself: our decomposition analysis showed that the relative increase in gout burden was larger in women than in men across all metrics (DALYs: 177.7% vs 159.5%), consistent with national evidence of a faster relative rise in overweight and obesity among middle-aged Chinese women,32 which would raise the female BMI-attributable fraction irrespective of any hormonal mechanism. Sex-specific genetic architecture may contribute further, since polymorphisms in ABCG2 are more strongly associated with serum urate in males, whereas SLC2A9 variants exhibit stronger associations in females.^[26]^ Sex differences in healthcare seeking, diagnostic ascertainment, and the reference exposure distributions used within the GBD comparative risk assessment could also generate an apparent crossover in PAF without any corresponding difference in underlying biology. Disentangling these explanations will require individual-level cohort data with documented menopausal status, serial anthropometry, and measured serum urate. Clinically, women remain particularly vulnerable to delayed diagnosis because gout in this group frequently presents in atypical joints such as the ankles, knees, wrists, or fingers rather than the classical podagra, often leading to misclassification as osteoarthritis or rheumatoid arthritis.^[7]^ Together, these findings raise the concern that the rising female burden documented here may be both underestimated by current surveillance systems and inadequately addressed by existing clinical pathways.

The Das Gupta decomposition revealed that population growth contributed approximately 70 to 73 percent of the absolute change in gout burden between 1990 and 2023, with epidemiological change accounting for 19 to 24 percent and population aging for 6 to 8 percent. The modest aging contribution is an expected consequence of the narrow 20-year age window examined here, within which the scope for shifts in age structure is inherently limited. While demographic momentum is the proximate quantitative driver, the policy-relevant component is the epidemiological change, since this is the only contribution amenable to modification through primary prevention. The substantial epidemiological component, which was particularly pronounced in men, indicates that age-specific gout risk has itself intensified meaningfully beyond what would be expected from purely demographic shifts – a finding that mirrors broader evidence on the metabolic transformation of the Chinese population over the same period.^[32]^ The disproportionately larger relative increases observed in women further suggest that female-targeted preventive strategies may have lagged behind those for men.

These findings carry several actionable implications for clinical practice and public health policy. Among the 2 attributable risk factors quantified for gout in GBD 2023, the increasing predominance of high BMI places weight management – encompassing the promotion of plant-forward dietary patterns, restriction of sugar-sweetened beverages, structured physical activity, and judicious use of pharmacological adjuncts where indicated – at the center of population-level gout prevention. Modeling work from China suggests that a transition to optimal dietary patterns could prevent up to 70% of the projected overweight and obesity burden by 2031, with potential national medical-cost savings of approximately 16.5%, an order of magnitude that, by extension, could substantially attenuate the gout trajectory we have projected.^[32]^ The BAPC projections indicate that prevalence will remain high, at approximately 6.34 million cases by 2035, underscoring the continuing need for intensified prevention. The rising female burden, particularly in the age strata spanning the menopausal transition, supports sex-disaggregated screening strategies and heightened diagnostic vigilance in middle-aged women. From a clinical management perspective, the 2024 update of the Chinese Guidelines for Diagnosis and Treatment of Hyperuricemia and Gout reaffirms long-term urate-lowering therapy as the cornerstone of treatment, with chronic management targeting serum urate concentrations between 180 and 300 μmol/L and emphasizing multidisciplinary management spanning rheumatology, endocrinology, nephrology, and primary care.^[33]^ However, real-world adherence to urate-lowering therapy in China remains suboptimal, and the persistent gap between guideline-concordant care and routine practice represents a critical bottleneck that mirrors the global treatment paradox in gout management.

## 5. Limitations

Several methodological considerations should temper the interpretation of our findings. First, this is an ecological analysis of modeled estimates rather than of primary individual-level data; GBD estimates are generated through Bayesian meta-regression of heterogeneous input data and inevitably carry uncertainty that may be amplified in demographic subgroups with sparser surveillance infrastructure, and associations observed at the population level cannot be interpreted as individual-level causal effects. Second, our analysis was deliberately restricted to adults aged 40 to 59 years. This restriction was specified a priori and permits a focused characterization of the stratum in which the burden is accumulating most rapidly, but it necessarily excludes adults aged 60 years and older, in whom the absolute gout burden is also substantial, in whom kidney dysfunction contributes a larger attributable fraction, and in whom comorbidity and polypharmacy complicate management. Our estimates of the aging contribution in the decomposition, and of the relative weight of BMI versus kidney dysfunction, therefore apply only to the 40 to 59-year window and should not be extrapolated to the total Chinese population; a parallel analysis of the 60+ population is a priority for future work. Third, only 2 attributable risk factors – high BMI and kidney dysfunction – are currently captured under the GBD framework for gout, meaning that the contributions of dietary purine load, alcohol consumption, fructose intake, diuretic exposure, and genetic susceptibility cannot be directly quantified, although these factors likely operate substantially through the BMI and renal pathways we modeled. Fourth, the sex crossover in BMI-attributable PAF from age 45 onward is reported here as an observation; because GBD data contain no information on menopausal status, adiposity trajectories, or hormone use, the menopausal interpretation we advance remains a hypothesis requiring confirmation in individual-level cohorts. Fifth, the apparent flattening of several indicators during 2020 to 2023 should be interpreted with caution, since healthcare-seeking behavior and case ascertainment were materially disrupted by the COVID-19 pandemic. Finally, BAPC projections to 2035 assume stability in age, period, and cohort effects beyond the observed window, and any disruptive change in the obesity trajectory, urate-lowering therapy uptake, or population age structure could alter the forecasted burden in either direction. Future research integrating primary surveillance data, province-level stratification, the 60+ population, and broader risk-factor coverage will be essential for refining the estimates presented here and for tracking the response of the Chinese gout epidemic to the preventive measures it now urgently demands.

## 6. Conclusion

Gout in China has evolved over the past 3 decades into a chronic, escalating, and metabolically driven epidemic, with prevalent cases among adults aged 40 to 59 years rising from 2.56 million in 1990 to 6.75 million in 2023 and projected to remain at a high level through 2035. Its attributable risk profile has shifted further toward metabolic risk, with high BMI becoming increasingly dominant relative to kidney dysfunction, while middle-aged women now bear a disproportionately rising and potentially underrecognized share of disease, a pattern for which the menopausal transition offers a plausible but as yet unconfirmed explanation. Containing this trajectory will require a shift from reactive clinical care toward upstream metabolic prevention, integrating population-level weight and dietary interventions, sex-sensitive screening pathways, and broader implementation of guideline-concordant urate-lowering therapy. The persistently high projected burden through 2035 represents an important opportunity for preventive action to reduce the future impact of this common metabolic disease.

## Acknowledgments

The authors thank the Institute for Health Metrics and Evaluation (IHME) and the Global Burden of Disease collaborators for making the GBD data and methodological resources publicly available. During manuscript preparation, ChatGPT (OpenAI) was used for language editing and technical formatting assistance. All AI-assisted output was reviewed and revised by the authors, who take full responsibility for the final content.

## Author contributions

**Formal analysis:** Hongwen Song, Zhuhui Duan, Zhiyu Wang, Qianqing Guo, Xiaoying Sun.

**Funding acquisition:** Zhaowei Zhou, Wei Zhu.

**Investigation:** Wei Xu.

**Methodology:** Quanwei He.

**Project administration:** Sensen Cheng.

**Resources:** Yueli Qi, Haishuo He.

**Software:** Zhaowei Zhou.

**Supervision:** Wei Zhu.

**Visualization:** Huan Nie.

**Validation:** Yaqiong Fan.

**Writing – original draft:** Chenyang Huang.

**Writing – review & editing:** Chenyang Huang.

















## References

[1] Dalbeth N Choi HK Joosten LAB. Gout. Nat Rev Dis Primers. 2019;5:69. 10.1038/s41572-019-0115-y 31558729

[2] Singh JA Gaffo A. Gout epidemiology and comorbidities. Semin Arthritis Rheum. 2020;50:S11–6. 10.1016/j.semarthrit.2020.04.008 32620196

[3] Im PK Kartsonaki C Kakkoura MG. Hyperuricemia, gout and the associated comorbidities in China: findings from a prospective study of 0.5 million adults. Lancet Reg Health West Pac. 2025;58:101572. 10.1016/j.lanwpc.2025.101572 PMC12167516 40519626

[4] Borghi C Agabiti-Rosei E Johnson RJ. Hyperuricaemia and gout in cardiovascular, metabolic and kidney disease. Eur J Intern Med. 2020;80:1–11. 10.1016/j.ejim.2020.07.006 32739239

[5] Xia Y Wu Q Wang H. Global, regional and national burden of gout, 1990-2017: a systematic analysis of the Global Burden of Disease Study. Rheumatology (Oxford). 2020;59:1529–38. 10.1093/rheumatology/kez476 31624843

[6] Zhu B Wang Y Zhou W. Trend dynamics of gout prevalence among the Chinese population, 1990-2019: a joinpoint and age-period-cohort analysis. Front Public Health. 2022;10:1008598. 10.3389/fpubh.2022.1008598 PMC9602928 36311630

[7] Dehlin M Jacobsson L Roddy E. Global epidemiology of gout: prevalence, incidence, treatment patterns and risk factors. Nat Rev Rheumatol. 2020;16:380–90. 10.1038/s41584-020-0441-1 32541923

[8] Xie S Xiao H Xu L Li G Zhang F Luo M. A comprehensive analysis of trends in the burden of gout in China and globally from 1990 to 2021. Sci Rep. 2025;15:3310. 10.1038/s41598-025-86090-z PMC11770106 39865102

[9] Qi H Chen H Dalbeth N. Two-decade trajectories of gout: a multicentre cohort study of 10 658 Chinese patients with gout. Rheumatology (Oxford). 2026;65:keaf555. 10.1093/rheumatology/keaf555 41137675

[10] Gao Q Cheng X Merriman TR. Trends in the manifestations of 9754 gout patients in a Chinese clinical center: a 10-year observational study. Joint Bone Spine. 2021;88:105078. 10.1016/j.jbspin.2020.09.010 32950704

[11] Tan X Liu X Shao H. Healthy China 2030: a vision for health care. Value Health Reg Issues. 2017;12:112–4. 10.1016/j.vhri.2017.04.001 28648308

[12] Zhong Y Li Y Hu C. Systematic analysis of gout burden among young adults in China from 1990 to 2021: findings from the global burden of disease study 2021. Front Public Health. 2025;13:1613801. 10.3389/fpubh.2025.1613801 PMC12183959 40552223

[13] GBD 2023 Disease and Injury and Risk Factor Collaborators. Burden of 375 diseases and injuries, risk-attributable burden of 88 risk factors, and healthy life expectancy in 204 countries and territories, including 660 subnational locations, 1990-2023: a systematic analysis for the Global Burden of Disease Study 2023. Lancet. 2025;406:1873–922. 10.1016/S0140-6736(25)01637-X PMC12535840 41092926

[14] GBD 2023 Causes of Death Collaborators. Global burden of 292 causes of death in 204 countries and territories and 660 subnational locations, 1990-2023: a systematic analysis for the Global Burden of Disease Study 2023. Lancet. 2025;406:1811–72. 10.1016/S0140-6736(25)01917-8 PMC12535838 41092928

[15] Kim HJ Fay MP Feuer EJ Midthune DN. Permutation tests for joinpoint regression with applications to cancer rates. Stat Med. 2000;19:335–51. 10.1002/(sici)1097-0258(20000215)19:3<335::aid-sim336>3.0.co;2-z 10649300

[16] Chevan A Sutherland M. Revisiting Das Gupta: refinement and extension of standardization and decomposition. Demography. 2009;46:429–49. 10.1353/dem.0.0060 PMC2831344 19771938

[17] Riebler A Held L. Projecting the future burden of cancer: Bayesian age-period-cohort analysis with integrated nested Laplace approximations. Biom J. 2017;59:531–49. 10.1002/bimj.201500263 28139001

[18] Stevens GA Alkema L Black RE. Guidelines for accurate and transparent health estimates reporting: the GATHER statement. PLoS Med. 2016;13:e1002056. 10.1371/journal.pmed.1002056 PMC4924581 27351744

[19] Song J Jin C Shan Z Teng W Li J. Prevalence and risk factors of hyperuricemia and gout: a cross-sectional survey from 31 provinces in Mainland China. J Transl Int Med. 2022;10:134–45. 10.2478/jtim-2022-0031 PMC9328039 35959454

[20] Chen Y Kang M Kim H Xu W Lee JE. Associations of dietary patterns with obesity and weight change for adults aged 18-65 years: evidence from the China Health and Nutrition Survey (CHNS). PLoS One. 2023;18:e0279625. 10.1371/journal.pone.0279625 PMC9876275 36696382

[21] Larsson SC Burgess S Michaelsson K. Genetic association between adiposity and gout: a Mendelian randomization study. Rheumatology (Oxford). 2018;57:2145–8. 10.1093/rheumatology/key229 PMC6697177 30085130

[22] Panlu K Zhou Z Huang L Ge L Wen C Lv H. Associations between obesity and hyperuricemia combing mendelian randomization with network pharmacology. Heliyon. 2024;10:e27074. 10.1016/j.heliyon.2024.e27074 PMC10951504 38509958

[23] Aune D Norat T Vatten LJ. Body mass index and the risk of gout: a systematic review and dose-response meta-analysis of prospective studies. Eur J Nutr. 2014;53:1591–601. 10.1007/s00394-014-0766-0 25209031

[24] Dalbeth N Gosling AL Gaffo A Abhishek A. Gout. Lancet. 2021;397:1843–55. 10.1016/S0140-6736(21)00569-9 33798500

[25] Yang Y Xian W Wu D. The role of obesity, type 2 diabetes, and metabolic factors in gout: a Mendelian randomization study. Front Endocrinol (Lausanne). 2022;13:917056. 10.3389/fendo.2022.917056 PMC9388832 35992130

[26] Halperin Kuhns VL Woodward OM. Sex differences in urate handling. Int J Mol Sci. 2020;21:4269. 10.3390/ijms21124269 PMC7349092 32560040

[27] Howard AG Attard SM Herring AH Wang H Du S Gordon-Larsen P. Socioeconomic gradients in the Westernization of diet in China over 20 years. SSM Popul Health. 2021;16:100943. 10.1016/j.ssmph.2021.100943 PMC8526760 34703875

[28] Xu J Lei M Xu D. Trends in the global burden of gout attributable to kidney dysfunction, 1990-2021: a population-based analysis. Front Endocrinol (Lausanne). 2025;16:1677340. 10.3389/fendo.2025.1677340 PMC12583099 41195208

[29] Hak AE Curhan GC Grodstein F Choi HK. Menopause, postmenopausal hormone use and risk of incident gout. Ann Rheum Dis. 2010;69:1305–9. 10.1136/ard.2009.109884 PMC3142742 19592386

[30] Hak AE Choi HK. Menopause, postmenopausal hormone use and serum uric acid levels in US women--the Third National Health and Nutrition Examination Survey. Arthritis Res Ther. 2008;10:R116. 10.1186/ar2519 PMC2592803 18822120

[31] Wang M Kartsonaki C Guo Y. Factors related to age at natural menopause in China: results from the China Kadoorie Biobank. Menopause. 2021;28:1130–42. 10.1097/GME.0000000000001829 PMC8462451 34342284

[32] Zhuang P Liu X Ao Y. Impact of dietary transitions on overweight and obesity burden and future trends among Chinese adults. npj Sci Food. 2025;9:257. 10.1038/s41538-025-00615-4 PMC12661000 41271819

[33] Sun M Lyu Z Wang C. 2024 update of Chinese guidelines for diagnosis and treatment of hyperuricemia and gout part i: recommendations for general patients. Int J Rheum Dis. 2025;28:e70375. 10.1111/1756-185x.70375 PMC12280528 40692263

