# Supplementary material for: Trends, decomposition, and projections of gout burden among middle-aged adults in China: GBD 2023
Source: Medicine (Baltimore). 2026 Oct 9;105(41):e51086. doi: 10.1097/MD.0000000000051086 (PMC13657502; doi:10.1097/MD.0000000000051086)
Supplement: Supplementary file 1 [file medi-105-e51086-s002.docx]

| **Supplementary Table S1：Projections of Gout Incidence Number in China (1990–2035)** | | | | | |
| --- | --- | --- | --- | --- | --- |
| **Year** | **Sex** | **Measure** | **Pred_val** | **Pred_low** | **Pred_up** |
| 1990 | Male | Incidence | 383352.8499 | 379946.3456 | 386759.3542 |
| 1991 | Male | Incidence | 385355.9792 | 381945.4178 | 388766.5407 |
| 1992 | Male | Incidence | 388789.1928 | 385363.9957 | 392214.3899 |
| 1993 | Male | Incidence | 394569.4369 | 391118.9115 | 398019.9623 |
| 1994 | Male | Incidence | 403532.8491 | 400042.7935 | 407022.9046 |
| 1995 | Male | Incidence | 414267.2986 | 410731.4321 | 417803.1651 |
| 1996 | Male | Incidence | 427117.8711 | 423527.2792 | 430708.463 |
| 1997 | Male | Incidence | 440543.1142 | 436895.7813 | 444190.4471 |
| 1998 | Male | Incidence | 453935.853 | 450233.2221 | 457638.4839 |
| 1999 | Male | Incidence | 468255.2571 | 464494.4384 | 472016.0759 |
| 2000 | Male | Incidence | 482656.6049 | 478840.605 | 486472.6048 |
| 2001 | Male | Incidence | 500174.2214 | 496290.3142 | 504058.1286 |
| 2002 | Male | Incidence | 521668.94 | 517700.2785 | 525637.6014 |
| 2003 | Male | Incidence | 546905.1275 | 542837.7749 | 550972.4801 |
| 2004 | Male | Incidence | 576136.4527 | 571956.7523 | 580316.1531 |
| 2005 | Male | Incidence | 605335.1601 | 601050.6296 | 609619.6906 |
| 2006 | Male | Incidence | 633382.6163 | 629000.3786 | 637764.854 |
| 2007 | Male | Incidence | 661029.9458 | 656551.8869 | 665508.0048 |
| 2008 | Male | Incidence | 690687.5278 | 686106.6168 | 695268.4387 |
| 2009 | Male | Incidence | 714344.293 | 709683.1723 | 719005.4136 |
| 2010 | Male | Incidence | 743948.6577 | 739192.6475 | 748704.6678 |
| 2011 | Male | Incidence | 774996.8254 | 770144.2721 | 779849.3786 |
| 2012 | Male | Incidence | 810515.8674 | 805553.3488 | 815478.386 |
| 2013 | Male | Incidence | 844627.0253 | 839558.7875 | 849695.2631 |
| 2014 | Male | Incidence | 872782.1056 | 867629.3245 | 877934.8867 |
| 2015 | Male | Incidence | 892150.7398 | 886944.4621 | 897357.0175 |
| 2016 | Male | Incidence | 906884.1524 | 901641.3222 | 912126.9827 |
| 2017 | Male | Incidence | 923802.6638 | 918517.818 | 929087.5095 |
| 2018 | Male | Incidence | 944061.4421 | 938724.8751 | 949398.0091 |
| 2019 | Male | Incidence | 964606.6265 | 959218.8113 | 969994.4418 |
| 2020 | Male | Incidence | 981273.0446 | 975846.623 | 986699.4662 |
| 2021 | Male | Incidence | 990787.2379 | 985342.8178 | 996231.658 |
| 2022 | Male | Incidence | 991106.652 | 985668.2938 | 996545.0103 |
| 2023 | Male | Incidence | 977023.5209 | 971625.0353 | 982422.0064 |
| 2024 | Male | Incidence | 1008894.63 | 967962.693 | 1049826.567 |
| 2025 | Male | Incidence | 999658.1445 | 947609.4472 | 1051706.842 |
| 2026 | Male | Incidence | 989406.9682 | 922096.5494 | 1056717.387 |
| 2027 | Male | Incidence | 979982.7638 | 894238.4888 | 1065727.039 |
| 2028 | Male | Incidence | 971289.942 | 864577.9319 | 1078001.952 |
| 2029 | Male | Incidence | 962327.5604 | 832578.8115 | 1092076.309 |
| 2030 | Male | Incidence | 953355.4109 | 798909.9255 | 1107800.896 |
| 2031 | Male | Incidence | 944571.0329 | 764016.1505 | 1125125.915 |
| 2032 | Male | Incidence | 936126.6831 | 728203.3386 | 1144050.028 |
| 2033 | Male | Incidence | 927969.5673 | 691562.2779 | 1164376.857 |
| 2034 | Male | Incidence | 920205.4522 | 654199.5186 | 1186211.386 |
| 2035 | Male | Incidence | 913124.5355 | 616334.687 | 1209914.384 |
| 1990 | Female | Incidence | 103438.018 | 101695.6965 | 105180.3395 |
| 1991 | Female | Incidence | 105980.3275 | 104221.7568 | 107738.8982 |
| 1992 | Female | Incidence | 108568.6199 | 106788.2224 | 110349.0175 |
| 1993 | Female | Incidence | 111241.9786 | 109438.3086 | 113045.6485 |
| 1994 | Female | Incidence | 114289.5061 | 112459.4508 | 116119.5614 |
| 1995 | Female | Incidence | 117258.3645 | 115403.5296 | 119113.1993 |
| 1996 | Female | Incidence | 120132.1187 | 118253.6439 | 122010.5935 |
| 1997 | Female | Incidence | 122485.8306 | 120588.321 | 124383.3402 |
| 1998 | Female | Incidence | 124476.6064 | 122563.7168 | 126389.496 |
| 1999 | Female | Incidence | 127114.977 | 125182.1141 | 129047.8398 |
| 2000 | Female | Incidence | 130102.2511 | 128147.8151 | 132056.687 |
| 2001 | Female | Incidence | 135117.8663 | 133126.2829 | 137109.4498 |
| 2002 | Female | Incidence | 142262.5789 | 140217.7893 | 144307.3685 |
| 2003 | Female | Incidence | 151149.7617 | 149039.5068 | 153260.0167 |
| 2004 | Female | Incidence | 161540.4531 | 159356.2546 | 163724.6515 |
| 2005 | Female | Incidence | 171614.8499 | 169362.8814 | 173866.8185 |
| 2006 | Female | Incidence | 181560.6264 | 179243.8083 | 183877.4446 |
| 2007 | Female | Incidence | 191574.4136 | 189192.953 | 193955.8743 |
| 2008 | Female | Incidence | 202202.8848 | 199754.0827 | 204651.6869 |
| 2009 | Female | Incidence | 210010.1924 | 207512.4697 | 212507.9151 |
| 2010 | Female | Incidence | 218471.9718 | 215925.1486 | 221018.795 |
| 2011 | Female | Incidence | 224690.9271 | 222108.5395 | 227273.3147 |
| 2012 | Female | Incidence | 230330.6924 | 227715.196 | 232946.1887 |
| 2013 | Female | Incidence | 234940.7225 | 232296.6464 | 237584.7986 |
| 2014 | Female | Incidence | 238634.3563 | 235968.1001 | 241300.6126 |
| 2015 | Female | Incidence | 242068.4371 | 239384.3589 | 244752.5152 |
| 2016 | Female | Incidence | 246894.854 | 244187.9526 | 249601.7555 |
| 2017 | Female | Incidence | 254079.8016 | 251338.7451 | 256820.8582 |
| 2018 | Female | Incidence | 263246.993 | 260462.2313 | 266031.7548 |
| 2019 | Female | Incidence | 272523.6279 | 269696.3499 | 275350.9058 |
| 2020 | Female | Incidence | 279691.6486 | 276834.4251 | 282548.8722 |
| 2021 | Female | Incidence | 283727.0452 | 280856.5242 | 286597.5661 |
| 2022 | Female | Incidence | 284736.6992 | 281866.8352 | 287606.5633 |
| 2023 | Female | Incidence | 280835.8837 | 277984.7015 | 283687.066 |
| 2024 | Female | Incidence | 292325.775 | 279001.8458 | 305649.7043 |
| 2025 | Female | Incidence | 289707.8982 | 273123.2923 | 306292.5041 |
| 2026 | Female | Incidence | 286701.7595 | 265652.3682 | 307751.1509 |
| 2027 | Female | Incidence | 284084.0441 | 257611.0274 | 310557.0608 |
| 2028 | Female | Incidence | 281844.4491 | 249158.4183 | 314530.48 |
| 2029 | Female | Incidence | 279522.3482 | 239982.9797 | 319061.7167 |
| 2030 | Female | Incidence | 277152.7487 | 230254.7549 | 324050.7425 |
| 2031 | Female | Incidence | 274788.6092 | 220108.2929 | 329468.9255 |
| 2032 | Female | Incidence | 272497.7341 | 209658.9807 | 335336.4874 |
| 2033 | Female | Incidence | 270339.1051 | 198992.375 | 341685.8351 |
| 2034 | Female | Incidence | 268385.2798 | 188159.7166 | 348610.843 |
| 2035 | Female | Incidence | 266689.3394 | 177192.6445 | 356186.0343 |
| 1990 | Both | Incidence | 486790.8679 | 481642.0421 | 491939.6937 |
| 1991 | Both | Incidence | 491336.3067 | 486167.1745 | 496505.4389 |
| 1992 | Both | Incidence | 497357.8127 | 492152.2181 | 502563.4074 |
| 1993 | Both | Incidence | 505811.4154 | 500557.2201 | 511065.6107 |
| 1994 | Both | Incidence | 517822.3552 | 512502.2443 | 523142.466 |
| 1995 | Both | Incidence | 531525.6631 | 526134.9617 | 536916.3644 |
| 1996 | Both | Incidence | 547249.9898 | 541780.9231 | 552719.0564 |
| 1997 | Both | Incidence | 563028.9448 | 557484.1023 | 568573.7873 |
| 1998 | Both | Incidence | 578412.4594 | 572796.9389 | 584027.9798 |
| 1999 | Both | Incidence | 595370.2341 | 589676.5525 | 601063.9157 |
| 2000 | Both | Incidence | 612758.856 | 606988.4202 | 618529.2918 |
| 2001 | Both | Incidence | 635292.0878 | 629416.5971 | 641167.5784 |
| 2002 | Both | Incidence | 663931.5189 | 657918.0678 | 669944.9699 |
| 2003 | Both | Incidence | 698054.8892 | 691877.2816 | 704232.4967 |
| 2004 | Both | Incidence | 737676.9058 | 731313.0069 | 744040.8046 |
| 2005 | Both | Incidence | 776950.01 | 770413.511 | 783486.5091 |
| 2006 | Both | Incidence | 814943.2427 | 808244.1868 | 821642.2986 |
| 2007 | Both | Incidence | 852604.3594 | 845744.8398 | 859463.879 |
| 2008 | Both | Incidence | 892890.4126 | 885860.6995 | 899920.1256 |
| 2009 | Both | Incidence | 924354.4854 | 917195.6421 | 931513.3287 |
| 2010 | Both | Incidence | 962420.6295 | 955117.7961 | 969723.4628 |
| 2011 | Both | Incidence | 999687.7525 | 992252.8116 | 1007122.693 |
| 2012 | Both | Incidence | 1040846.56 | 1033268.545 | 1048424.575 |
| 2013 | Both | Incidence | 1079567.748 | 1071855.434 | 1087280.062 |
| 2014 | Both | Incidence | 1111416.462 | 1103597.425 | 1119235.499 |
| 2015 | Both | Incidence | 1134219.177 | 1126328.821 | 1142109.533 |
| 2016 | Both | Incidence | 1153779.006 | 1145829.275 | 1161728.738 |
| 2017 | Both | Incidence | 1177882.465 | 1169856.563 | 1185908.368 |
| 2018 | Both | Incidence | 1207308.435 | 1199187.106 | 1215429.764 |
| 2019 | Both | Incidence | 1237130.254 | 1228915.161 | 1245345.348 |
| 2020 | Both | Incidence | 1260964.693 | 1252681.048 | 1269248.338 |
| 2021 | Both | Incidence | 1274514.283 | 1266199.342 | 1282829.224 |
| 2022 | Both | Incidence | 1275843.351 | 1267535.129 | 1284151.574 |
| 2023 | Both | Incidence | 1257859.405 | 1249609.737 | 1266109.072 |
| 2024 | Both | Incidence | 1301220.405 | 1246964.539 | 1355476.271 |
| 2025 | Both | Incidence | 1289366.043 | 1220732.74 | 1357999.346 |
| 2026 | Both | Incidence | 1276108.728 | 1187748.918 | 1364468.538 |
| 2027 | Both | Incidence | 1264066.808 | 1151849.516 | 1376284.1 |
| 2028 | Both | Incidence | 1253134.391 | 1113736.35 | 1392532.432 |
| 2029 | Both | Incidence | 1241849.909 | 1072561.791 | 1411138.026 |
| 2030 | Both | Incidence | 1230508.16 | 1029164.68 | 1431851.639 |
| 2031 | Both | Incidence | 1219359.642 | 984124.4434 | 1454594.841 |
| 2032 | Both | Incidence | 1208624.417 | 937862.3193 | 1479386.515 |
| 2033 | Both | Incidence | 1198308.672 | 890554.6529 | 1506062.692 |
| 2034 | Both | Incidence | 1188590.732 | 842359.2352 | 1534822.229 |
| 2035 | Both | Incidence | 1179813.875 | 793527.3314 | 1566100.418 |
