# Supplementary material for: Trends, decomposition, and projections of gout burden among middle-aged adults in China: GBD 2023
Source: Medicine (Baltimore). 2026 Oct 9;105(41):e51086. doi: 10.1097/MD.0000000000051086 (PMC13657502; doi:10.1097/MD.0000000000051086)
Supplement: Supplementary file 2 [file medi-105-e51086-s003.docx]

| **Supplementary Table S2：Projections of Gout Incidence Rate in China (1990–2035)** | | | | | |
| --- | --- | --- | --- | --- | --- |
| **Year** | **Sex** | **Measure** | **Pred_val** | **Pred_low** | **Pred_up** |
| 1990 | Male | Incidence | 348.4425245 | 347.3451044 | 349.5399445 |
| 1991 | Male | Incidence | 341.0536234 | 339.9897921 | 342.1174546 |
| 1992 | Male | Incidence | 334.9096931 | 333.869143 | 335.9502432 |
| 1993 | Male | Incidence | 330.2454404 | 329.224842 | 331.2660387 |
| 1994 | Male | Incidence | 327.2927389 | 326.2911458 | 328.2943321 |
| 1995 | Male | Incidence | 326.2654087 | 325.2771454 | 327.253672 |
| 1996 | Male | Incidence | 327.1905361 | 326.2128603 | 328.168212 |
| 1997 | Male | Incidence | 329.5576323 | 328.5875304 | 330.5277342 |
| 1998 | Male | Incidence | 332.7740628 | 331.8091059 | 333.7390197 |
| 1999 | Male | Incidence | 336.254928 | 335.2962806 | 337.2135754 |
| 2000 | Male | Incidence | 339.3992901 | 338.4452643 | 340.3533159 |
| 2001 | Male | Incidence | 342.5860805 | 341.6398948 | 343.5322662 |
| 2002 | Male | Incidence | 346.3584606 | 345.4226148 | 347.2943063 |
| 2003 | Male | Incidence | 350.3730039 | 349.4492245 | 351.2967833 |
| 2004 | Male | Incidence | 354.264656 | 353.356574 | 355.172738 |
| 2005 | Male | Incidence | 357.6903738 | 356.7941419 | 358.5866057 |
| 2006 | Male | Incidence | 360.7701241 | 359.8846567 | 361.6555915 |
| 2007 | Male | Incidence | 363.973595 | 363.0988287 | 364.8483614 |
| 2008 | Male | Incidence | 367.4585693 | 366.5962558 | 368.3208828 |
| 2009 | Male | Incidence | 371.3781986 | 370.5218767 | 372.2345204 |
| 2010 | Male | Incidence | 375.8856745 | 375.035194 | 376.736155 |
| 2011 | Male | Incidence | 382.6806759 | 381.8305471 | 383.5308047 |
| 2012 | Male | Incidence | 392.1653752 | 391.3131647 | 393.0175858 |
| 2013 | Male | Incidence | 402.4973174 | 401.6418706 | 403.3527641 |
| 2014 | Male | Incidence | 411.8190843 | 410.9585347 | 412.6796338 |
| 2015 | Male | Incidence | 418.2727011 | 417.4066375 | 419.1387647 |
| 2016 | Male | Incidence | 422.5201474 | 421.6499625 | 423.3903323 |
| 2017 | Male | Incidence | 426.3485274 | 425.4767729 | 427.220282 |
| 2018 | Male | Incidence | 429.5917688 | 428.721993 | 430.4615446 |
| 2019 | Male | Incidence | 432.0716379 | 431.2046431 | 432.9386327 |
| 2020 | Male | Incidence | 433.6159111 | 432.7505029 | 434.4813194 |
| 2021 | Male | Incidence | 434.5187211 | 433.6522469 | 435.3851952 |
| 2022 | Male | Incidence | 434.3593605 | 433.4898811 | 435.2288398 |
| 2023 | Male | Incidence | 430.4020734 | 429.5294948 | 431.274652 |
| 2024 | Male | Incidence | 430.5560305 | 418.5515381 | 442.5605228 |
| 2025 | Male | Incidence | 429.0550483 | 411.1618985 | 446.9481981 |
| 2026 | Male | Incidence | 427.4989671 | 402.1866547 | 452.8112795 |
| 2027 | Male | Incidence | 425.8902514 | 392.023143 | 459.7573598 |
| 2028 | Male | Incidence | 424.2397943 | 380.8593172 | 467.6202714 |
| 2029 | Male | Incidence | 422.5958976 | 368.8340083 | 476.357787 |
| 2030 | Male | Incidence | 420.9717548 | 356.081176 | 485.8623335 |
| 2031 | Male | Incidence | 419.3648192 | 342.6737681 | 496.0558702 |
| 2032 | Male | Incidence | 417.760399 | 328.6415279 | 506.8792701 |
| 2033 | Male | Incidence | 416.1490421 | 313.9944564 | 518.3036278 |
| 2034 | Male | Incidence | 414.5541372 | 298.7597598 | 530.3485146 |
| 2035 | Male | Incidence | 412.984208 | 282.9860715 | 542.9823445 |
| 1990 | Female | Incidence | 104.7082655 | 104.0809318 | 105.3355993 |
| 1991 | Female | Incidence | 104.1061007 | 103.5017324 | 104.7104689 |
| 1992 | Female | Incidence | 103.5098935 | 102.9162527 | 104.1035342 |
| 1993 | Female | Incidence | 102.9276188 | 102.342803 | 103.5124345 |
| 1994 | Female | Incidence | 102.3739739 | 101.7989007 | 102.9490471 |
| 1995 | Female | Incidence | 101.8614236 | 101.2946965 | 102.4281507 |
| 1996 | Female | Incidence | 101.2449377 | 100.6874193 | 101.8024561 |
| 1997 | Female | Incidence | 100.4776816 | 99.92937605 | 101.0259871 |
| 1998 | Female | Incidence | 99.72062852 | 99.18057434 | 100.2606827 |
| 1999 | Female | Incidence | 99.14429226 | 98.61338488 | 99.67519964 |
| 2000 | Female | Incidence | 98.931106 | 98.40650239 | 99.45570961 |
| 2001 | Female | Incidence | 99.58254434 | 99.06406133 | 100.1010274 |
| 2002 | Female | Incidence | 101.2152596 | 100.7019226 | 101.7285965 |
| 2003 | Female | Incidence | 103.3852303 | 102.8763061 | 103.8941545 |
| 2004 | Female | Incidence | 105.6395226 | 105.136749 | 106.1422961 |
| 2005 | Female | Incidence | 107.5161675 | 107.0181177 | 108.0142172 |
| 2006 | Female | Incidence | 109.1683579 | 108.6755317 | 109.6611841 |
| 2007 | Female | Incidence | 110.9293783 | 110.4414409 | 111.4173156 |
| 2008 | Female | Incidence | 112.6137454 | 112.1321376 | 113.0953532 |
| 2009 | Female | Incidence | 114.0230556 | 113.5445899 | 114.5015213 |
| 2010 | Female | Incidence | 114.9529134 | 114.4790372 | 115.4267896 |
| 2011 | Female | Incidence | 115.4877866 | 115.0173263 | 115.9582469 |
| 2012 | Female | Incidence | 115.8870047 | 115.4206034 | 116.3534061 |
| 2013 | Female | Incidence | 116.2534678 | 115.7909769 | 116.7159586 |
| 2014 | Female | Incidence | 116.6908555 | 116.2303711 | 117.1513399 |
| 2015 | Female | Incidence | 117.2988512 | 116.8382427 | 117.7594597 |
| 2016 | Female | Incidence | 118.4432978 | 117.9813516 | 118.9052441 |
| 2017 | Female | Incidence | 120.1577527 | 119.694861 | 120.6206444 |
| 2018 | Female | Incidence | 122.0274417 | 121.5651662 | 122.4897172 |
| 2019 | Female | Incidence | 123.6313796 | 123.1700886 | 124.0926706 |
| 2020 | Female | Incidence | 124.5521089 | 124.0916648 | 125.0125531 |
| 2021 | Female | Incidence | 124.9015026 | 124.4409698 | 125.3620354 |
| 2022 | Female | Incidence | 124.8861605 | 124.4240604 | 125.3482605 |
| 2023 | Female | Incidence | 123.6952746 | 123.2286863 | 124.161863 |
| 2024 | Female | Incidence | 124.2143519 | 120.3784083 | 128.0502954 |
| 2025 | Female | Incidence | 123.9654765 | 118.3769391 | 129.5540139 |
| 2026 | Female | Incidence | 123.694247 | 115.8940272 | 131.4944668 |
| 2027 | Female | Incidence | 123.3987503 | 113.0447327 | 133.7527679 |
| 2028 | Female | Incidence | 123.0810792 | 109.8830049 | 136.2791536 |
| 2029 | Female | Incidence | 122.7517822 | 106.4462414 | 139.057323 |
| 2030 | Female | Incidence | 122.4044357 | 102.7674264 | 142.0414449 |
| 2031 | Female | Incidence | 122.0366794 | 98.86821596 | 145.2051427 |
| 2032 | Female | Incidence | 121.6492959 | 94.76193547 | 148.5366562 |
| 2033 | Female | Incidence | 121.247076 | 90.45694219 | 152.0372098 |
| 2034 | Female | Incidence | 120.8430335 | 85.96517685 | 155.7208902 |
| 2035 | Female | Incidence | 120.440513 | 81.30245732 | 159.5785686 |
| 1990 | Both | Incidence | 233.021588 | 230.5567901 | 235.486386 |
| 1991 | Both | Incidence | 228.5562648 | 226.1512747 | 230.9612548 |
| 1992 | Both | Incidence | 224.8022266 | 222.4482199 | 227.1562334 |
| 1993 | Both | Incidence | 221.9151344 | 219.6064295 | 224.2238393 |
| 1994 | Both | Incidence | 219.9545235 | 217.6901189 | 222.2189281 |
| 1995 | Both | Incidence | 219.0155742 | 216.785768 | 221.2453803 |
| 1996 | Both | Incidence | 219.0038664 | 216.8047714 | 221.2029615 |
| 1997 | Both | Incidence | 219.6683261 | 217.4943477 | 221.8423044 |
| 1998 | Both | Incidence | 220.8114457 | 218.6567227 | 222.9661687 |
| 1999 | Both | Incidence | 222.080436 | 219.9468671 | 224.2140049 |
| 2000 | Both | Incidence | 223.4202879 | 221.3025019 | 225.5380738 |
| 2001 | Both | Incidence | 225.1353065 | 223.0379071 | 227.2327059 |
| 2002 | Both | Incidence | 227.621098 | 225.546826 | 229.69537 |
| 2003 | Both | Incidence | 230.4640514 | 228.4157612 | 232.5123416 |
| 2004 | Both | Incidence | 233.3746088 | 231.3584262 | 235.3907915 |
| 2005 | Both | Incidence | 235.8560106 | 233.8662348 | 237.8457865 |
| 2006 | Both | Incidence | 237.9733736 | 236.0089977 | 239.9377495 |
| 2007 | Both | Incidence | 240.214782 | 238.2745924 | 242.1549716 |
| 2008 | Both | Incidence | 242.580519 | 240.6676604 | 244.4933775 |
| 2009 | Both | Incidence | 245.0959235 | 243.1972356 | 246.9946115 |
| 2010 | Both | Incidence | 247.7681385 | 245.8851976 | 249.6510795 |
| 2011 | Both | Incidence | 251.4987057 | 249.6219277 | 253.3754838 |
| 2012 | Both | Incidence | 256.4839734 | 254.6099757 | 258.3579711 |
| 2013 | Both | Incidence | 261.8224779 | 259.9487354 | 263.6962204 |
| 2014 | Both | Incidence | 266.6539425 | 264.7757088 | 268.5321763 |
| 2015 | Both | Incidence | 270.0961546 | 268.2106976 | 271.9816116 |
| 2016 | Both | Incidence | 272.6739717 | 270.7813128 | 274.5666306 |
| 2017 | Both | Incidence | 275.3246998 | 273.427689 | 277.2217107 |
| 2018 | Both | Incidence | 277.7679915 | 275.8726853 | 279.6632977 |
| 2019 | Both | Incidence | 279.7509853 | 277.8593056 | 281.6426651 |
| 2020 | Both | Incidence | 280.9767674 | 279.0876711 | 282.8658637 |
| 2021 | Both | Incidence | 281.6373819 | 279.7463176 | 283.5284463 |
| 2022 | Both | Incidence | 281.6023518 | 279.7052713 | 283.4994323 |
| 2023 | Both | Incidence | 279.0923916 | 277.1928901 | 280.991893 |
| 2024 | Both | Incidence | 278.7728291 | 267.0657329 | 290.4799253 |
| 2025 | Both | Incidence | 277.9277443 | 263.029652 | 292.8258366 |
| 2026 | Both | Incidence | 277.0674795 | 257.7736422 | 296.3613168 |
| 2027 | Both | Incidence | 276.1754778 | 251.5486282 | 300.8023275 |
| 2028 | Both | Incidence | 275.236938 | 244.5077041 | 305.9661718 |
| 2029 | Both | Incidence | 274.3091915 | 236.8024377 | 311.8159454 |
| 2030 | Both | Incidence | 273.4461481 | 228.5874075 | 318.3048886 |
| 2031 | Both | Incidence | 272.6903102 | 219.9577052 | 325.4229151 |
| 2032 | Both | Incidence | 272.0783623 | 210.9652911 | 333.1914334 |
| 2033 | Both | Incidence | 271.6063119 | 201.6017759 | 341.6108479 |
| 2034 | Both | Incidence | 271.2830763 | 191.8580076 | 350.708145 |
| 2035 | Both | Incidence | 271.1447567 | 181.7623981 | 360.5271153 |
