# Supplementary material for: Trends, decomposition, and projections of gout burden among middle-aged adults in China: GBD 2023
Source: Medicine (Baltimore). 2026 Oct 9;105(41):e51086. doi: 10.1097/MD.0000000000051086 (PMC13657502; doi:10.1097/MD.0000000000051086)
Supplement: Supplementary file 3 [file medi-105-e51086-s004.docx]

| **Supplementary Table S3 Projections of Gout DALYs Number in China (1990–2035)** | | | | | | |
| --- | --- | --- | --- | --- | --- | --- |
|  | **Year** | **Sex** | **Measure** | **Pred_val** | **Pred_low** | **Pred_up** |
|  | 1990 | Male | DALYs | 65315.289 | 63957.264 | 66673.31465 |
|  | 1991 | Male | DALYs | 65741.938 | 64391.455 | 67092.42113 |
|  | 1992 | Male | DALYs | 66128.266 | 64775.692 | 67480.84097 |
|  | 1993 | Male | DALYs | 66993.4 | 65631.857 | 68354.9431 |
|  | 1994 | Male | DALYs | 68497.762 | 67119.944 | 69875.57878 |
|  | 1995 | Male | DALYs | 70271.114 | 68874.621 | 71667.60806 |
|  | 1996 | Male | DALYs | 72511.901 | 71091.873 | 73931.92855 |
|  | 1997 | Male | DALYs | 74854.55 | 73409.845 | 76299.25564 |
|  | 1998 | Male | DALYs | 77344.869 | 75874.85 | 78814.88716 |
|  | 1999 | Male | DALYs | 79936.863 | 78440.792 | 81432.93409 |
|  | 2000 | Male | DALYs | 82484.465 | 80964.766 | 84004.16376 |
|  | 2001 | Male | DALYs | 86250.765 | 84695.171 | 87806.35828 |
|  | 2002 | Male | DALYs | 90452.171 | 88856.371 | 92047.97039 |
|  | 2003 | Male | DALYs | 95445.619 | 93802.129 | 97089.10908 |
|  | 2004 | Male | DALYs | 100957.17 | 99261.777 | 102652.5681 |
|  | 2005 | Male | DALYs | 106323.31 | 104580.98 | 108065.6332 |
|  | 2006 | Male | DALYs | 111183.35 | 109399.74 | 112966.9569 |
|  | 2007 | Male | DALYs | 115762.64 | 113940.5 | 117584.7711 |
|  | 2008 | Male | DALYs | 120589.33 | 118726.31 | 122452.3495 |
|  | 2009 | Male | DALYs | 124364.24 | 122469.67 | 126258.806 |
|  | 2010 | Male | DALYs | 129006.38 | 127075.41 | 130937.3498 |
|  | 2011 | Male | DALYs | 134075.94 | 132106.57 | 136045.3152 |
|  | 2012 | Male | DALYs | 139713.44 | 137701.85 | 141725.0367 |
|  | 2013 | Male | DALYs | 145362.59 | 143308.43 | 147416.746 |
|  | 2014 | Male | DALYs | 149627.18 | 147541.37 | 151712.9895 |
|  | 2015 | Male | DALYs | 153046.16 | 150937.48 | 155154.839 |
|  | 2016 | Male | DALYs | 155799.53 | 153674.66 | 157924.4054 |
|  | 2017 | Male | DALYs | 158655.03 | 156513.26 | 160796.8116 |
|  | 2018 | Male | DALYs | 163010.69 | 160841.62 | 165179.7565 |
|  | 2019 | Male | DALYs | 166584.48 | 164394.02 | 168774.941 |
|  | 2020 | Male | DALYs | 169551.66 | 167344.39 | 171758.9407 |
|  | 2021 | Male | DALYs | 171304.81 | 169089.38 | 173520.2306 |
|  | 2022 | Male | DALYs | 171958.5 | 169739.72 | 174177.2726 |
|  | 2023 | Male | DALYs | 169697.22 | 167486.31 | 171908.1298 |
|  | 2024 | Male | DALYs | 175153.94 | 168226.48 | 182081.3992 |
|  | 2025 | Male | DALYs | 173636.57 | 164686.03 | 182587.114 |
|  | 2026 | Male | DALYs | 171930.4 | 160214.63 | 183646.1753 |
|  | 2027 | Male | DALYs | 170368.86 | 155325.75 | 185411.9633 |
|  | 2028 | Male | DALYs | 168929.23 | 150109.06 | 187749.4051 |
|  | 2029 | Male | DALYs | 167432.91 | 144466.13 | 190399.702 |
|  | 2030 | Male | DALYs | 165938.59 | 138525.7 | 193351.4815 |
|  | 2031 | Male | DALYs | 164500.68 | 132380.8 | 196620.5672 |
|  | 2032 | Male | DALYs | 163168.73 | 126099.73 | 200237.7314 |
|  | 2033 | Male | DALYs | 161939.83 | 119696.96 | 204182.7003 |
|  | 2034 | Male | DALYs | 160825.28 | 113178.5 | 208472.0553 |
|  | 2035 | Male | DALYs | 159710.73 | 106660.04 | 212761.4103 |
|  | 1990 | Female | DALYs | 16720.271 | 16074.196 | 17366.34567 |
|  | 1991 | Female | DALYs | 17219.443 | 16574.327 | 17864.5593 |
|  | 1992 | Female | DALYs | 17679.344 | 17027.438 | 18331.24909 |
|  | 1993 | Female | DALYs | 18212.37 | 17549.983 | 18874.75738 |
|  | 1994 | Female | DALYs | 18759.539 | 18086.001 | 19433.07659 |
|  | 1995 | Female | DALYs | 19236.14 | 18553.068 | 19919.21148 |
|  | 1996 | Female | DALYs | 19700.691 | 19008.451 | 20392.93062 |
|  | 1997 | Female | DALYs | 20130.396 | 19429.947 | 20830.84567 |
|  | 1998 | Female | DALYs | 20534.511 | 19826.25 | 21242.77152 |
|  | 1999 | Female | DALYs | 20967.963 | 20251.978 | 21683.94895 |
|  | 2000 | Female | DALYs | 21514.597 | 20788.778 | 22240.41636 |
|  | 2001 | Female | DALYs | 22310.193 | 21570.048 | 23050.33906 |
|  | 2002 | Female | DALYs | 23340.754 | 22581.914 | 24099.59396 |
|  | 2003 | Female | DALYs | 24690.799 | 23907.43 | 25474.16828 |
|  | 2004 | Female | DALYs | 26239.839 | 25428.537 | 27051.14152 |
|  | 2005 | Female | DALYs | 27748.984 | 26912.015 | 28585.95261 |
|  | 2006 | Female | DALYs | 29297.314 | 28434.754 | 30159.87485 |
|  | 2007 | Female | DALYs | 30855.727 | 29967.285 | 31744.16838 |
|  | 2008 | Female | DALYs | 32563.275 | 31647.348 | 33479.20263 |
|  | 2009 | Female | DALYs | 33796.72 | 32861.114 | 34732.32678 |
|  | 2010 | Female | DALYs | 35296.483 | 34338.536 | 36254.4309 |
|  | 2011 | Female | DALYs | 36452.975 | 35478.523 | 37427.42758 |
|  | 2012 | Female | DALYs | 37449.601 | 36460.664 | 38438.53698 |
|  | 2013 | Female | DALYs | 38422.241 | 37418.584 | 39425.89886 |
|  | 2014 | Female | DALYs | 39158.325 | 38143.647 | 40173.00325 |
|  | 2015 | Female | DALYs | 39921.587 | 38896.748 | 40946.42653 |
|  | 2016 | Female | DALYs | 40717.921 | 39683.735 | 41752.10691 |
|  | 2017 | Female | DALYs | 41915.602 | 40867.256 | 42963.94737 |
|  | 2018 | Female | DALYs | 43306.647 | 42241.526 | 44371.76885 |
|  | 2019 | Female | DALYs | 44988.771 | 43903.74 | 46073.80168 |
|  | 2020 | Female | DALYs | 46155.811 | 45058.221 | 47253.40077 |
|  | 2021 | Female | DALYs | 46871.011 | 45766.506 | 47975.5169 |
|  | 2022 | Female | DALYs | 47139.065 | 46031.24 | 48246.89121 |
|  | 2023 | Female | DALYs | 46484.195 | 45373.9 | 47594.4899 |
|  | 2024 | Female | DALYs | 48353.679 | 46175.787 | 50531.57093 |
|  | 2025 | Female | DALYs | 47928.706 | 45264.329 | 50593.08343 |
|  | 2026 | Female | DALYs | 47437.597 | 44110.014 | 50765.18056 |
|  | 2027 | Female | DALYs | 47014.801 | 42879.929 | 51149.67373 |
|  | 2028 | Female | DALYs | 46658.907 | 41596.166 | 51721.6477 |
|  | 2029 | Female | DALYs | 46291.366 | 40202.248 | 52380.48384 |
|  | 2030 | Female | DALYs | 45918.06 | 38724.822 | 53111.29868 |
|  | 2031 | Female | DALYs | 45546.727 | 37184.534 | 53908.92126 |
|  | 2032 | Female | DALYs | 45188.159 | 35599.979 | 54776.3389 |
|  | 2033 | Female | DALYs | 44853.301 | 33986.381 | 55720.22125 |
|  | 2034 | Female | DALYs | 44552.467 | 32351.192 | 56753.74272 |
|  | 2035 | Female | DALYs | 44251.634 | 30716.003 | 57787.26419 |
|  | 1990 | Both | DALYs | 82035.56 | 80031.46 | 84039.66032 |
|  | 1991 | Both | DALYs | 82961.381 | 80965.781 | 84956.98042 |
|  | 1992 | Both | DALYs | 83807.61 | 81803.13 | 85812.09006 |
|  | 1993 | Both | DALYs | 85205.77 | 83181.839 | 87229.70049 |
|  | 1994 | Both | DALYs | 87257.3 | 85205.945 | 89308.65537 |
|  | 1995 | Both | DALYs | 89507.254 | 87427.689 | 91586.81954 |
|  | 1996 | Both | DALYs | 92212.592 | 90100.324 | 94324.85917 |
|  | 1997 | Both | DALYs | 94984.947 | 92839.792 | 97130.10131 |
|  | 1998 | Both | DALYs | 97879.379 | 95701.1 | 100057.6587 |
|  | 1999 | Both | DALYs | 100904.83 | 98692.769 | 103116.883 |
|  | 2000 | Both | DALYs | 103999.06 | 101753.54 | 106244.5801 |
|  | 2001 | Both | DALYs | 108560.96 | 106265.22 | 110856.6973 |
|  | 2002 | Both | DALYs | 113792.92 | 111438.28 | 116147.5643 |
|  | 2003 | Both | DALYs | 120136.42 | 117709.56 | 122563.2774 |
|  | 2004 | Both | DALYs | 127197.01 | 124690.31 | 129703.7096 |
|  | 2005 | Both | DALYs | 134072.29 | 131492.99 | 136651.5858 |
|  | 2006 | Both | DALYs | 140480.66 | 137834.49 | 143126.8318 |
|  | 2007 | Both | DALYs | 146618.36 | 143907.79 | 149328.9395 |
|  | 2008 | Both | DALYs | 153152.61 | 150373.66 | 155931.5522 |
|  | 2009 | Both | DALYs | 158160.96 | 155330.78 | 160991.1328 |
|  | 2010 | Both | DALYs | 164302.86 | 161413.95 | 167191.7807 |
|  | 2011 | Both | DALYs | 170528.92 | 167585.1 | 173472.7428 |
|  | 2012 | Both | DALYs | 177163.04 | 174162.51 | 180163.5737 |
|  | 2013 | Both | DALYs | 183784.83 | 180727.01 | 186842.6448 |
|  | 2014 | Both | DALYs | 188785.51 | 185685.02 | 191885.9928 |
|  | 2015 | Both | DALYs | 192967.75 | 189834.23 | 196101.2656 |
|  | 2016 | Both | DALYs | 196517.45 | 193358.4 | 199676.5124 |
|  | 2017 | Both | DALYs | 200570.64 | 197380.51 | 203760.759 |
|  | 2018 | Both | DALYs | 206317.33 | 203083.14 | 209551.5254 |
|  | 2019 | Both | DALYs | 211573.25 | 208297.76 | 214848.7427 |
|  | 2020 | Both | DALYs | 215707.47 | 212402.61 | 219012.3415 |
|  | 2021 | Both | DALYs | 218175.82 | 214855.89 | 221495.7476 |
|  | 2022 | Both | DALYs | 219097.56 | 215770.96 | 222424.1638 |
|  | 2023 | Both | DALYs | 216181.42 | 212860.21 | 219502.6197 |
|  | 2024 | Both | DALYs | 223507.62 | 214402.27 | 232612.9702 |
|  | 2025 | Both | DALYs | 221565.28 | 209950.36 | 233180.1974 |
|  | 2026 | Both | DALYs | 219368 | 204324.65 | 234411.3559 |
|  | 2027 | Both | DALYs | 217383.66 | 198205.68 | 236561.6371 |
|  | 2028 | Both | DALYs | 215588.14 | 191705.23 | 239471.0528 |
|  | 2029 | Both | DALYs | 213724.28 | 184668.37 | 242780.1858 |
|  | 2030 | Both | DALYs | 211856.65 | 177250.52 | 246462.7802 |
|  | 2031 | Both | DALYs | 210047.41 | 169565.33 | 250529.4885 |
|  | 2032 | Both | DALYs | 208356.89 | 161699.71 | 255014.0703 |
|  | 2033 | Both | DALYs | 206793.13 | 153683.34 | 259902.9216 |
|  | 2034 | Both | DALYs | 205377.75 | 145529.69 | 265225.798 |
|  | 2035 | Both | DALYs | 203962.36 | 137376.05 | 270548.6744 |
