# Supplementary material for: Trends, decomposition, and projections of gout burden among middle-aged adults in China: GBD 2023
Source: Medicine (Baltimore). 2026 Oct 9;105(41):e51086. doi: 10.1097/MD.0000000000051086 (PMC13657502; doi:10.1097/MD.0000000000051086)
Supplement: Supplementary file 4 [file medi-105-e51086-s005.docx]

| **Supplementary Table S4 Projections of Gout DALYs Rate in China (1990–2035)** | | | | | |
| --- | --- | --- | --- | --- | --- |
| **Year** | **Sex** | **Measure** | **Pred_val** | **Pred_low** | **Pred_up** |
| 1990 | Male | DALYs | 59.414678 | 58.974818 | 59.85453897 |
| 1991 | Male | DALYs | 58.260563 | 57.850367 | 58.6707601 |
| 1992 | Male | DALYs | 57.066906 | 56.667171 | 57.46664143 |
| 1993 | Male | DALYs | 56.202087 | 55.810039 | 56.59413481 |
| 1994 | Male | DALYs | 55.707244 | 55.322072 | 56.09241548 |
| 1995 | Male | DALYs | 55.528747 | 55.148318 | 55.90917622 |
| 1996 | Male | DALYs | 55.74115 | 55.364046 | 56.11825329 |
| 1997 | Male | DALYs | 56.191265 | 55.816144 | 56.56638487 |
| 1998 | Male | DALYs | 56.871409 | 56.497207 | 57.245612 |
| 1999 | Male | DALYs | 57.540279 | 57.167457 | 57.91310097 |
| 2000 | Male | DALYs | 58.120487 | 57.749061 | 58.49191312 |
| 2001 | Male | DALYs | 59.150435 | 58.779775 | 59.52109496 |
| 2002 | Male | DALYs | 60.099056 | 59.730739 | 60.46737327 |
| 2003 | Male | DALYs | 61.176186 | 60.810351 | 61.54202156 |
| 2004 | Male | DALYs | 62.115198 | 61.753527 | 62.47686861 |
| 2005 | Male | DALYs | 62.892075 | 62.533872 | 63.25027807 |
| 2006 | Male | DALYs | 63.424131 | 63.069675 | 63.7785867 |
| 2007 | Male | DALYs | 63.846394 | 63.496107 | 64.19668225 |
| 2008 | Male | DALYs | 64.26769 | 63.9222 | 64.61317927 |
| 2009 | Male | DALYs | 64.749641 | 64.406586 | 65.09269515 |
| 2010 | Male | DALYs | 65.255118 | 64.914693 | 65.59554346 |
| 2011 | Male | DALYs | 66.264793 | 65.924608 | 66.60497782 |
| 2012 | Male | DALYs | 67.625372 | 67.284711 | 67.96603259 |
| 2013 | Male | DALYs | 69.26776 | 68.925665 | 69.60985504 |
| 2014 | Male | DALYs | 70.58257 | 70.238762 | 70.92637833 |
| 2015 | Male | DALYs | 71.710971 | 71.364806 | 72.05713554 |
| 2016 | Male | DALYs | 72.496432 | 72.148716 | 72.84414849 |
| 2017 | Male | DALYs | 73.092163 | 72.743999 | 73.4403279 |
| 2018 | Male | DALYs | 73.988202 | 73.64011 | 74.33629468 |
| 2019 | Male | DALYs | 74.361578 | 74.014893 | 74.70826256 |
| 2020 | Male | DALYs | 74.620949 | 74.275044 | 74.96685403 |
| 2021 | Male | DALYs | 74.75459 | 74.408586 | 75.10059436 |
| 2022 | Male | DALYs | 74.992335 | 74.644165 | 75.34050517 |
| 2023 | Male | DALYs | 74.36028 | 74.004229 | 74.71633157 |
| 2024 | Male | DALYs | 74.429727 | 72.406339 | 76.45311444 |
| 2025 | Male | DALYs | 74.24135 | 71.15302 | 77.32967933 |
| 2026 | Male | DALYs | 74.041452 | 69.619289 | 78.4636149 |
| 2027 | Male | DALYs | 73.828233 | 67.871063 | 79.78540216 |
| 2028 | Male | DALYs | 73.60383 | 65.939974 | 81.26768585 |
| 2029 | Male | DALYs | 73.379193 | 63.851465 | 82.906921 |
| 2030 | Male | DALYs | 73.156056 | 61.628348 | 84.6837633 |
| 2031 | Male | DALYs | 72.935686 | 59.28477 | 86.58660308 |
| 2032 | Male | DALYs | 72.718857 | 56.828427 | 88.60928685 |
| 2033 | Male | DALYs | 72.502536 | 54.259441 | 90.74563123 |
| 2034 | Male | DALYs | 72.291954 | 51.582734 | 93.00117433 |
| 2035 | Male | DALYs | 72.135647 | 50.268588 | 95.00277355 |
| 1990 | Female | DALYs | 16.930764 | 16.700019 | 17.16150807 |
| 1991 | Female | DALYs | 16.926192 | 16.721409 | 17.13097446 |
| 1992 | Female | DALYs | 16.873455 | 16.674895 | 17.07201408 |
| 1993 | Female | DALYs | 16.879025 | 16.68232 | 17.07573077 |
| 1994 | Female | DALYs | 16.8355 | 16.641223 | 17.02977647 |
| 1995 | Female | DALYs | 16.758784 | 16.566926 | 16.95064189 |
| 1996 | Female | DALYs | 16.653209 | 16.464148 | 16.84227028 |
| 1997 | Female | DALYs | 16.563522 | 16.377122 | 16.74992147 |
| 1998 | Female | DALYs | 16.492102 | 16.307701 | 16.67650239 |
| 1999 | Female | DALYs | 16.385681 | 16.204045 | 16.56731687 |
| 2000 | Female | DALYs | 16.382585 | 16.202464 | 16.56270557 |
| 2001 | Female | DALYs | 16.445982 | 16.267562 | 16.62440236 |
| 2002 | Female | DALYs | 16.603271 | 16.426504 | 16.78003824 |
| 2003 | Female | DALYs | 16.87755 | 16.701716 | 17.05338396 |
| 2004 | Female | DALYs | 17.151972 | 16.977367 | 17.32657681 |
| 2005 | Female | DALYs | 17.385273 | 17.211703 | 17.5588425 |
| 2006 | Female | DALYs | 17.618739 | 17.446334 | 17.7911434 |
| 2007 | Female | DALYs | 17.880273 | 17.708627 | 18.05192013 |
| 2008 | Female | DALYs | 18.147242 | 17.976868 | 18.31761568 |
| 2009 | Female | DALYs | 18.357519 | 18.187652 | 18.52738669 |
| 2010 | Female | DALYs | 18.578117 | 18.408895 | 18.74733821 |
| 2011 | Female | DALYs | 18.737427 | 18.5688 | 18.9060543 |
| 2012 | Female | DALYs | 18.842476 | 18.674803 | 19.01014963 |
| 2013 | Female | DALYs | 19.011254 | 18.843965 | 19.17854318 |
| 2014 | Female | DALYs | 19.142164 | 18.975019 | 19.30930859 |
| 2015 | Female | DALYs | 19.335959 | 19.16821 | 19.50370882 |
| 2016 | Female | DALYs | 19.524384 | 19.356112 | 19.69265682 |
| 2017 | Female | DALYs | 19.800153 | 19.631428 | 19.96887686 |
| 2018 | Female | DALYs | 20.046819 | 19.87832 | 20.21531809 |
| 2019 | Female | DALYs | 20.364013 | 20.19534 | 20.53268573 |
| 2020 | Female | DALYs | 20.491265 | 20.323019 | 20.65951061 |
| 2021 | Female | DALYs | 20.562975 | 20.394821 | 20.73112947 |
| 2022 | Female | DALYs | 20.593838 | 20.424106 | 20.76356952 |
| 2023 | Female | DALYs | 20.402523 | 20.222384 | 20.5826614 |
| 2024 | Female | DALYs | 20.490003 | 19.896648 | 21.08335736 |
| 2025 | Female | DALYs | 20.461187 | 19.598355 | 21.32401899 |
| 2026 | Female | DALYs | 20.428847 | 19.230338 | 21.62735643 |
| 2027 | Female | DALYs | 20.393008 | 18.808727 | 21.9772899 |
| 2028 | Female | DALYs | 20.354695 | 18.34139 | 22.36799941 |
| 2029 | Female | DALYs | 20.315872 | 17.833783 | 22.79796009 |
| 2030 | Female | DALYs | 20.274913 | 17.289907 | 23.25991861 |
| 2031 | Female | DALYs | 20.230226 | 16.711628 | 23.74882393 |
| 2032 | Female | DALYs | 20.180616 | 16.099614 | 24.26161825 |
| 2033 | Female | DALYs | 20.126432 | 15.454732 | 24.79813289 |
| 2034 | Female | DALYs | 20.069999 | 14.779138 | 25.36086058 |
| 2035 | Female | DALYs | 20.013566 | 14.103545 | 25.92358827 |
| 1990 | Both | DALYs | 39.298498 | 38.338748 | 40.25824835 |
| 1991 | Both | DALYs | 38.639029 | 37.709945 | 39.56811342 |
| 1992 | Both | DALYs | 37.945616 | 37.038359 | 38.85287355 |
| 1993 | Both | DALYs | 37.467104 | 36.57678 | 38.35742844 |
| 1994 | Both | DALYs | 37.161827 | 36.287554 | 38.03610002 |
| 1995 | Both | DALYs | 37.004209 | 36.142669 | 37.86574882 |
| 1996 | Both | DALYs | 37.029122 | 36.178429 | 37.87981473 |
| 1997 | Both | DALYs | 37.185026 | 36.34263 | 38.02742075 |
| 1998 | Both | DALYs | 37.475503 | 36.638542 | 38.3124642 |
| 1999 | Both | DALYs | 37.72522 | 36.895436 | 38.55500309 |
| 2000 | Both | DALYs | 37.991514 | 37.16686 | 38.81616818 |
| 2001 | Both | DALYs | 38.510615 | 37.69104 | 39.3301901 |
| 2002 | Both | DALYs | 39.031572 | 38.219466 | 39.8436788 |
| 2003 | Both | DALYs | 39.670121 | 38.865559 | 40.47468443 |
| 2004 | Both | DALYs | 40.253054 | 39.458784 | 41.04732437 |
| 2005 | Both | DALYs | 40.731397 | 39.946005 | 41.51678967 |
| 2006 | Both | DALYs | 41.070189 | 40.293967 | 41.84641036 |
| 2007 | Both | DALYs | 41.367205 | 40.600131 | 42.13427844 |
| 2008 | Both | DALYs | 41.669652 | 40.912966 | 42.42633888 |
| 2009 | Both | DALYs | 41.986927 | 41.235843 | 42.73801075 |
| 2010 | Both | DALYs | 42.337845 | 41.592674 | 43.08301713 |
| 2011 | Both | DALYs | 42.930697 | 42.187471 | 43.67392235 |
| 2012 | Both | DALYs | 43.666689 | 42.924699 | 44.40867953 |
| 2013 | Both | DALYs | 44.567242 | 43.824403 | 45.31008067 |
| 2014 | Both | DALYs | 45.279453 | 44.534826 | 46.02407998 |
| 2015 | Both | DALYs | 45.924668 | 45.176245 | 46.67309147 |
| 2016 | Both | DALYs | 46.391387 | 45.639998 | 47.14277536 |
| 2017 | Both | DALYs | 46.805109 | 46.052235 | 47.55798277 |
| 2018 | Both | DALYs | 47.357786 | 46.604634 | 48.11093834 |
| 2019 | Both | DALYs | 47.690866 | 46.938875 | 48.44285655 |
| 2020 | Both | DALYs | 47.88221 | 47.1313 | 48.63311966 |
| 2021 | Both | DALYs | 47.989702 | 47.237901 | 48.74150405 |
| 2022 | Both | DALYs | 48.135008 | 47.378696 | 48.89132042 |
| 2023 | Both | DALYs | 47.733777 | 46.971885 | 48.49566891 |
| 2024 | Both | DALYs | 47.699367 | 45.735995 | 49.66273975 |
| 2025 | Both | DALYs | 47.596753 | 45.078982 | 50.11452363 |
| 2026 | Both | DALYs | 47.490661 | 44.211128 | 50.77019335 |
| 2027 | Both | DALYs | 47.377053 | 43.174899 | 51.57920597 |
| 2028 | Both | DALYs | 47.253775 | 41.996385 | 52.51116546 |
| 2029 | Both | DALYs | 47.132248 | 40.702207 | 53.56228811 |
| 2030 | Both | DALYs | 47.021253 | 39.318074 | 54.72443192 |
| 2031 | Both | DALYs | 46.928472 | 37.860289 | 55.99665624 |
| 2032 | Both | DALYs | 46.861286 | 36.338249 | 57.38432338 |
| 2033 | Both | DALYs | 46.81798 | 34.749325 | 58.88663584 |
| 2034 | Both | DALYs | 46.800763 | 33.092084 | 60.50944229 |
| 2035 | Both | DALYs | 46.783546 | 31.434844 | 62.13224874 |
