# Supplementary material for: Trends, decomposition, and projections of gout burden among middle-aged adults in China: GBD 2023
Source: Medicine (Baltimore). 2026 Oct 9;105(41):e51086. doi: 10.1097/MD.0000000000051086 (PMC13657502; doi:10.1097/MD.0000000000051086)
Supplement: Supplementary file 5 [file medi-105-e51086-s006.docx]

| **Supplementary Table S5 Projections of Gout Prevalence Number in China (1990–2035)** | | | | | |
| --- | --- | --- | --- | --- | --- |
| **Year** | **Sex** | **Measure** | **Pred_val** | **Pred_low** | **Pred_up** |
| 1990 | Male | Prevalence | 2039603.614 | 2031706.702 | 2047500.526 |
| 1991 | Male | Prevalence | 2044458.167 | 2036552.598 | 2052363.736 |
| 1992 | Male | Prevalence | 2058034.257 | 2050101.075 | 2065967.44 |
| 1993 | Male | Prevalence | 2084419.683 | 2076434.355 | 2092405.012 |
| 1994 | Male | Prevalence | 2129113.665 | 2121041.516 | 2137185.814 |
| 1995 | Male | Prevalence | 2183504.334 | 2175329.621 | 2191679.047 |
| 1996 | Male | Prevalence | 2251301.299 | 2243000.454 | 2259602.144 |
| 1997 | Male | Prevalence | 2324540.783 | 2316105.768 | 2332975.797 |
| 1998 | Male | Prevalence | 2400099.78 | 2391530.43 | 2408669.131 |
| 1999 | Male | Prevalence | 2483887.195 | 2475172.713 | 2492601.676 |
| 2000 | Male | Prevalence | 2569193.25 | 2560338.306 | 2578048.194 |
| 2001 | Male | Prevalence | 2676406.255 | 2667373.352 | 2685439.158 |
| 2002 | Male | Prevalence | 2809931.73 | 2800674.169 | 2819189.292 |
| 2003 | Male | Prevalence | 2964148.021 | 2954633.168 | 2973662.873 |
| 2004 | Male | Prevalence | 3137522.181 | 3127723.38 | 3147320.983 |
| 2005 | Male | Prevalence | 3302893.958 | 3292840.93 | 3312946.987 |
| 2006 | Male | Prevalence | 3453695.425 | 3443417.468 | 3463973.382 |
| 2007 | Male | Prevalence | 3597152.125 | 3586661.238 | 3607643.012 |
| 2008 | Male | Prevalence | 3748981.337 | 3738265.333 | 3759697.341 |
| 2009 | Male | Prevalence | 3866735.642 | 3855848.827 | 3877622.458 |
| 2010 | Male | Prevalence | 4018805.933 | 4007711.79 | 4029900.075 |
| 2011 | Male | Prevalence | 4178078.739 | 4166773.841 | 4189383.638 |
| 2012 | Male | Prevalence | 4359062.286 | 4347518.327 | 4370606.245 |
| 2013 | Male | Prevalence | 4532115.755 | 4520342.533 | 4543888.976 |
| 2014 | Male | Prevalence | 4675700.961 | 4663743.803 | 4687658.119 |
| 2015 | Male | Prevalence | 4777805.421 | 4765728.656 | 4789882.185 |
| 2016 | Male | Prevalence | 4859960.532 | 4847797.715 | 4872123.348 |
| 2017 | Male | Prevalence | 4956186.432 | 4943923.309 | 4968449.556 |
| 2018 | Male | Prevalence | 5072715.68 | 5060328.275 | 5085103.085 |
| 2019 | Male | Prevalence | 5191955.524 | 5179444.233 | 5204466.815 |
| 2020 | Male | Prevalence | 5290003.853 | 5277398.035 | 5302609.671 |
| 2021 | Male | Prevalence | 5350442.258 | 5337787.455 | 5363097.06 |
| 2022 | Male | Prevalence | 5361422.001 | 5348773.762 | 5374070.24 |
| 2023 | Male | Prevalence | 5294544.342 | 5281985.17 | 5307103.514 |
| 2024 | Male | Prevalence | 5461100.992 | 5254671.04 | 5667530.943 |
| 2025 | Male | Prevalence | 5411321.908 | 5142661.19 | 5679982.626 |
| 2026 | Male | Prevalence | 5355207.382 | 5001591.72 | 5708823.044 |
| 2027 | Male | Prevalence | 5303609.247 | 4847982.896 | 5759235.598 |
| 2028 | Male | Prevalence | 5255617.819 | 4684421.766 | 5826813.872 |
| 2029 | Male | Prevalence | 5205538.082 | 4507691.543 | 5903384.621 |
| 2030 | Male | Prevalence | 5155769.445 | 4322295.631 | 5989243.259 |
| 2031 | Male | Prevalence | 5108243.591 | 4131299.094 | 6085188.089 |
| 2032 | Male | Prevalence | 5064489.841 | 3936767.878 | 6192211.803 |
| 2033 | Male | Prevalence | 5024407.022 | 3739078.104 | 6309735.94 |
| 2034 | Male | Prevalence | 4988089.321 | 3538184.602 | 6437994.04 |
| 2035 | Male | Prevalence | 4956736.981 | 3334857.62 | 6578616.343 |
| 1990 | Female | Prevalence | 519156.4033 | 515202.7342 | 523110.0723 |
| 1991 | Female | Prevalence | 533505.7359 | 529498.2334 | 537513.2384 |
| 1992 | Female | Prevalence | 547918.4727 | 543854.0107 | 551982.9348 |
| 1993 | Female | Prevalence | 562384.1959 | 558262.4082 | 566505.9836 |
| 1994 | Female | Prevalence | 578583.1468 | 574398.1885 | 582768.1051 |
| 1995 | Female | Prevalence | 594078.9653 | 589835.6186 | 598322.3121 |
| 1996 | Female | Prevalence | 609060.7209 | 604762.4805 | 613358.9612 |
| 1997 | Female | Prevalence | 621356.3691 | 617014.4076 | 625698.3306 |
| 1998 | Female | Prevalence | 631800.2803 | 627423.4261 | 636177.1345 |
| 1999 | Female | Prevalence | 645811.9782 | 641390.077 | 650233.8793 |
| 2000 | Female | Prevalence | 661273.0186 | 656803.4607 | 665742.5765 |
| 2001 | Female | Prevalence | 686191.1597 | 681641.7624 | 690740.5569 |
| 2002 | Female | Prevalence | 720182.4718 | 715521.9786 | 724842.965 |
| 2003 | Female | Prevalence | 761666.1854 | 756870.7635 | 766461.6074 |
| 2004 | Female | Prevalence | 810551.7018 | 805602.8359 | 815500.5676 |
| 2005 | Female | Prevalence | 858253.8482 | 853162.7661 | 863344.9302 |
| 2006 | Female | Prevalence | 905998.9441 | 900769.5238 | 911228.3644 |
| 2007 | Female | Prevalence | 954350.4919 | 948982.2009 | 959718.7829 |
| 2008 | Female | Prevalence | 1006789.696 | 1001274.044 | 1012305.348 |
| 2009 | Female | Prevalence | 1046392.926 | 1040767.373 | 1052018.479 |
| 2010 | Female | Prevalence | 1091273.809 | 1085533.024 | 1097014.594 |
| 2011 | Female | Prevalence | 1126452.958 | 1120623.249 | 1132282.668 |
| 2012 | Female | Prevalence | 1159405.97 | 1153491.396 | 1165320.544 |
| 2013 | Female | Prevalence | 1187268.466 | 1181279.08 | 1193257.851 |
| 2014 | Female | Prevalence | 1210209.727 | 1204160.775 | 1216258.68 |
| 2015 | Female | Prevalence | 1231324.101 | 1225226.943 | 1237421.258 |
| 2016 | Female | Prevalence | 1258628.742 | 1252475.036 | 1264782.448 |
| 2017 | Female | Prevalence | 1297254.493 | 1291021.438 | 1303487.548 |
| 2018 | Female | Prevalence | 1346181.259 | 1339848.208 | 1352514.31 |
| 2019 | Female | Prevalence | 1396299.714 | 1389868.607 | 1402730.821 |
| 2020 | Female | Prevalence | 1436612.724 | 1430109.418 | 1443116.031 |
| 2021 | Female | Prevalence | 1462395.134 | 1455852.929 | 1468937.339 |
| 2022 | Female | Prevalence | 1471991.95 | 1465444.558 | 1478539.342 |
| 2023 | Female | Prevalence | 1452730.183 | 1446232.664 | 1459227.702 |
| 2024 | Female | Prevalence | 1512296.522 | 1453586.227 | 1571006.816 |
| 2025 | Female | Prevalence | 1499480.045 | 1426092.851 | 1572867.238 |
| 2026 | Female | Prevalence | 1484248.672 | 1390833.983 | 1577663.36 |
| 2027 | Female | Prevalence | 1470861.378 | 1353164.876 | 1588557.879 |
| 2028 | Female | Prevalence | 1459174.23 | 1313692.371 | 1604656.088 |
| 2029 | Female | Prevalence | 1446803.82 | 1270692.08 | 1622915.56 |
| 2030 | Female | Prevalence | 1434232.284 | 1225254.989 | 1643209.579 |
| 2031 | Female | Prevalence | 1421954.448 | 1178217.17 | 1665691.725 |
| 2032 | Female | Prevalence | 1410481.008 | 1130257.728 | 1690704.287 |
| 2033 | Female | Prevalence | 1400058.398 | 1081720.651 | 1718396.146 |
| 2034 | Female | Prevalence | 1390917.545 | 1032743.872 | 1749091.218 |
| 2035 | Female | Prevalence | 1383307.346 | 983492.8607 | 1783121.831 |
| 1990 | Both | Prevalence | 2558760.017 | 2546909.436 | 2570610.598 |
| 1991 | Both | Prevalence | 2577963.903 | 2566050.832 | 2589876.974 |
| 1992 | Both | Prevalence | 2605952.73 | 2593955.086 | 2617950.374 |
| 1993 | Both | Prevalence | 2646803.879 | 2634696.763 | 2658910.996 |
| 1994 | Both | Prevalence | 2707696.812 | 2695439.704 | 2719953.919 |
| 1995 | Both | Prevalence | 2777583.3 | 2765165.24 | 2790001.359 |
| 1996 | Both | Prevalence | 2860362.02 | 2847762.934 | 2872961.105 |
| 1997 | Both | Prevalence | 2945897.152 | 2933120.176 | 2958674.127 |
| 1998 | Both | Prevalence | 3031900.061 | 3018953.856 | 3044846.265 |
| 1999 | Both | Prevalence | 3129699.173 | 3116562.79 | 3142835.556 |
| 2000 | Both | Prevalence | 3230466.269 | 3217141.767 | 3243790.771 |
| 2001 | Both | Prevalence | 3362597.414 | 3349015.114 | 3376179.715 |
| 2002 | Both | Prevalence | 3530114.202 | 3516196.148 | 3544032.257 |
| 2003 | Both | Prevalence | 3725814.206 | 3711503.931 | 3740124.48 |
| 2004 | Both | Prevalence | 3948073.883 | 3933326.216 | 3962821.55 |
| 2005 | Both | Prevalence | 4161147.806 | 4146003.696 | 4176291.917 |
| 2006 | Both | Prevalence | 4359694.369 | 4344186.992 | 4375201.746 |
| 2007 | Both | Prevalence | 4551502.617 | 4535643.439 | 4567361.795 |
| 2008 | Both | Prevalence | 4755771.033 | 4739539.377 | 4772002.689 |
| 2009 | Both | Prevalence | 4913128.568 | 4896616.199 | 4929640.937 |
| 2010 | Both | Prevalence | 5110079.742 | 5093244.814 | 5126914.669 |
| 2011 | Both | Prevalence | 5304531.698 | 5287397.089 | 5321666.306 |
| 2012 | Both | Prevalence | 5518468.255 | 5501009.723 | 5535926.788 |
| 2013 | Both | Prevalence | 5719384.22 | 5701621.614 | 5737146.827 |
| 2014 | Both | Prevalence | 5885910.688 | 5867904.578 | 5903916.798 |
| 2015 | Both | Prevalence | 6009129.521 | 5990955.599 | 6027303.443 |
| 2016 | Both | Prevalence | 6118589.273 | 6100272.751 | 6136905.795 |
| 2017 | Both | Prevalence | 6253440.925 | 6234944.747 | 6271937.104 |
| 2018 | Both | Prevalence | 6418896.939 | 6400176.482 | 6437617.395 |
| 2019 | Both | Prevalence | 6588255.238 | 6569312.84 | 6607197.636 |
| 2020 | Both | Prevalence | 6726616.577 | 6707507.452 | 6745725.702 |
| 2021 | Both | Prevalence | 6812837.391 | 6793640.384 | 6832034.399 |
| 2022 | Both | Prevalence | 6833413.951 | 6814218.32 | 6852609.582 |
| 2023 | Both | Prevalence | 6747274.525 | 6728217.834 | 6766331.216 |
| 2024 | Both | Prevalence | 6973397.513 | 6708257.267 | 7238537.759 |
| 2025 | Both | Prevalence | 6910801.953 | 6568754.041 | 7252849.864 |
| 2026 | Both | Prevalence | 6839456.053 | 6392425.702 | 7286486.404 |
| 2027 | Both | Prevalence | 6774470.625 | 6201147.772 | 7347793.477 |
| 2028 | Both | Prevalence | 6714792.048 | 5998114.137 | 7431469.96 |
| 2029 | Both | Prevalence | 6652341.903 | 5778383.624 | 7526300.182 |
| 2030 | Both | Prevalence | 6590001.729 | 5547550.62 | 7632452.838 |
| 2031 | Both | Prevalence | 6530198.039 | 5309516.264 | 7750879.814 |
| 2032 | Both | Prevalence | 6474970.848 | 5067025.606 | 7882916.09 |
| 2033 | Both | Prevalence | 6424465.42 | 4820798.755 | 8028132.086 |
| 2034 | Both | Prevalence | 6379006.866 | 4570928.474 | 8187085.258 |
| 2035 | Both | Prevalence | 6340044.327 | 4318350.48 | 8361738.174 |
