# Supplementary material for: Trends, decomposition, and projections of gout burden among middle-aged adults in China: GBD 2023
Source: Medicine (Baltimore). 2026 Oct 9;105(41):e51086. doi: 10.1097/MD.0000000000051086 (PMC13657502; doi:10.1097/MD.0000000000051086)
Supplement: Supplementary file 6 [file medi-105-e51086-s007.docx]

| **Supplementary Table S6 Projections of Gout Prevalence Rate in China (1990–2035)** | | | | | |
| --- | --- | --- | --- | --- | --- |
| **Year** | **Sex** | **Measure** | **Pred_val** | **Pred_low** | **Pred_up** |
| 1990 | Male | Prevalence | 1856.155428 | 1853.608273 | 1858.702583 |
| 1991 | Male | Prevalence | 1812.798513 | 1810.316412 | 1815.280615 |
| 1992 | Male | Prevalence | 1777.207853 | 1774.780528 | 1779.635179 |
| 1993 | Male | Prevalence | 1750.474614 | 1748.094564 | 1752.854664 |
| 1994 | Male | Prevalence | 1733.739825 | 1731.404442 | 1736.075208 |
| 1995 | Male | Prevalence | 1728.094357 | 1725.789614 | 1730.3991 |
| 1996 | Male | Prevalence | 1733.854416 | 1731.573822 | 1736.135011 |
| 1997 | Male | Prevalence | 1748.409452 | 1746.145694 | 1750.67321 |
| 1998 | Male | Prevalence | 1768.52257 | 1766.269696 | 1770.775445 |
| 1999 | Male | Prevalence | 1791.003398 | 1788.764412 | 1793.242384 |
| 2000 | Male | Prevalence | 1812.583521 | 1810.353624 | 1814.813419 |
| 2001 | Male | Prevalence | 1837.334667 | 1835.12018 | 1839.549153 |
| 2002 | Male | Prevalence | 1868.445331 | 1866.25037 | 1870.640291 |
| 2003 | Male | Prevalence | 1901.415141 | 1899.243199 | 1903.587083 |
| 2004 | Male | Prevalence | 1931.728094 | 1929.589312 | 1933.866877 |
| 2005 | Male | Prevalence | 1954.915757 | 1952.802875 | 1957.028639 |
| 2006 | Male | Prevalence | 1971.173418 | 1969.086527 | 1973.260309 |
| 2007 | Male | Prevalence | 1985.057106 | 1982.997774 | 1987.116438 |
| 2008 | Male | Prevalence | 1998.709804 | 1996.683395 | 2000.736214 |
| 2009 | Male | Prevalence | 2014.21142 | 2012.202651 | 2016.22019 |
| 2010 | Male | Prevalence | 2033.638058 | 2031.646269 | 2035.629847 |
| 2011 | Male | Prevalence | 2065.393148 | 2063.405264 | 2067.381033 |
| 2012 | Male | Prevalence | 2110.786313 | 2108.797036 | 2112.77559 |
| 2013 | Male | Prevalence | 2160.952873 | 2158.959378 | 2162.946369 |
| 2014 | Male | Prevalence | 2207.014756 | 2205.011812 | 2209.0177 |
| 2015 | Male | Prevalence | 2240.074417 | 2238.059682 | 2242.089153 |
| 2016 | Male | Prevalence | 2262.951175 | 2260.927293 | 2264.975057 |
| 2017 | Male | Prevalence | 2283.751027 | 2281.724505 | 2285.777549 |
| 2018 | Male | Prevalence | 2301.502737 | 2299.482519 | 2303.522956 |
| 2019 | Male | Prevalence | 2315.200883 | 2313.188985 | 2317.212781 |
| 2020 | Male | Prevalence | 2323.921352 | 2321.914625 | 2325.928078 |
| 2021 | Male | Prevalence | 2330.33449 | 2328.32561 | 2332.343371 |
| 2022 | Male | Prevalence | 2331.814593 | 2329.798599 | 2333.830586 |
| 2023 | Male | Prevalence | 2313.781105 | 2311.76195 | 2315.80026 |
| 2024 | Male | Prevalence | 2314.853159 | 2253.473143 | 2376.233175 |
| 2025 | Male | Prevalence | 2308.182493 | 2214.512804 | 2401.852181 |
| 2026 | Male | Prevalence | 2301.115144 | 2166.837209 | 2435.39308 |
| 2027 | Male | Prevalence | 2293.650932 | 2112.58856 | 2474.713305 |
| 2028 | Male | Prevalence | 2285.866897 | 2052.77342 | 2518.960374 |
| 2029 | Male | Prevalence | 2278.104155 | 1988.180693 | 2568.027617 |
| 2030 | Male | Prevalence | 2270.505405 | 1919.602496 | 2621.408315 |
| 2031 | Male | Prevalence | 2263.092758 | 1847.463805 | 2678.721711 |
| 2032 | Male | Prevalence | 2255.76494 | 1771.902273 | 2739.627607 |
| 2033 | Male | Prevalence | 2248.450402 | 1692.941177 | 2803.959626 |
| 2034 | Male | Prevalence | 2241.309973 | 1610.727279 | 2871.892666 |
| 2035 | Male | Prevalence | 2234.434945 | 1525.548464 | 2943.321427 |
| 1990 | Female | Prevalence | 526.0395517 | 524.6138584 | 527.4652451 |
| 1991 | Female | Prevalence | 524.9398614 | 523.5431249 | 526.3365979 |
| 1992 | Female | Prevalence | 523.6372067 | 522.2611825 | 525.013231 |
| 1993 | Female | Prevalence | 522.1473107 | 520.7897658 | 523.5048557 |
| 1994 | Female | Prevalence | 520.5313596 | 519.1948799 | 521.8678393 |
| 1995 | Female | Prevalence | 518.8412092 | 517.5227957 | 520.1596228 |
| 1996 | Female | Prevalence | 516.262653 | 514.9652252 | 517.5600807 |
| 1997 | Female | Prevalence | 512.587091 | 511.3113547 | 513.8628274 |
| 1998 | Female | Prevalence | 508.7445189 | 507.488712 | 510.0003259 |
| 1999 | Female | Prevalence | 505.7108197 | 504.478003 | 506.9436363 |
| 2000 | Female | Prevalence | 504.4605192 | 503.2439544 | 505.6770839 |
| 2001 | Female | Prevalence | 506.9145901 | 505.714914 | 508.1142663 |
| 2002 | Female | Prevalence | 513.2726921 | 512.0886826 | 514.4567017 |
| 2003 | Female | Prevalence | 521.811998 | 520.6424831 | 522.9815128 |
| 2004 | Female | Prevalence | 530.8218146 | 529.6710926 | 531.9725367 |
| 2005 | Female | Prevalence | 538.593158 | 537.4561784 | 539.7301376 |
| 2006 | Female | Prevalence | 545.7251919 | 544.602326 | 546.8480577 |
| 2007 | Female | Prevalence | 553.5578745 | 552.4482597 | 554.6674893 |
| 2008 | Female | Prevalence | 561.4319163 | 560.3385666 | 562.5252661 |
| 2009 | Female | Prevalence | 568.6376436 | 567.552117 | 569.7231702 |
| 2010 | Female | Prevalence | 574.4265342 | 573.3508657 | 575.5022026 |
| 2011 | Female | Prevalence | 579.1075932 | 578.037979 | 580.1772075 |
| 2012 | Female | Prevalence | 583.493426 | 582.4310794 | 584.5557727 |
| 2013 | Female | Prevalence | 587.8228658 | 586.7674418 | 588.8782898 |
| 2014 | Female | Prevalence | 592.3310374 | 591.2782562 | 593.3838186 |
| 2015 | Female | Prevalence | 597.2517068 | 596.197042 | 598.3063716 |
| 2016 | Female | Prevalence | 604.1191042 | 603.0606927 | 605.1775158 |
| 2017 | Female | Prevalence | 613.1753498 | 612.1152015 | 614.2354982 |
| 2018 | Female | Prevalence | 622.6803183 | 621.6228944 | 623.7377423 |
| 2019 | Female | Prevalence | 630.8760087 | 629.8222207 | 631.9297966 |
| 2020 | Female | Prevalence | 636.0069381 | 634.9557986 | 637.0580777 |
| 2021 | Female | Prevalence | 639.0963872 | 638.0444739 | 640.1483005 |
| 2022 | Female | Prevalence | 640.2888304 | 639.2331368 | 641.3445241 |
| 2023 | Female | Prevalence | 634.1923484 | 633.1338981 | 635.2507987 |
| 2024 | Female | Prevalence | 637.6757949 | 620.7436754 | 654.6079145 |
| 2025 | Female | Prevalence | 637.0063998 | 612.3214086 | 661.6913911 |
| 2026 | Female | Prevalence | 636.1851903 | 601.6952314 | 670.6751491 |
| 2027 | Female | Prevalence | 635.2002111 | 589.3732947 | 681.0271275 |
| 2028 | Female | Prevalence | 634.079297 | 575.6042322 | 692.5543619 |
| 2029 | Female | Prevalence | 632.9304768 | 560.5996821 | 705.2612714 |
| 2030 | Female | Prevalence | 631.7449706 | 544.5280589 | 718.9618823 |
| 2031 | Female | Prevalence | 630.5089069 | 527.4792045 | 733.5386094 |
| 2032 | Female | Prevalence | 629.2004786 | 509.4825345 | 748.9184227 |
| 2033 | Female | Prevalence | 627.8275779 | 490.5524639 | 765.1026919 |
| 2034 | Female | Prevalence | 626.4762889 | 470.7533365 | 782.1992412 |
| 2035 | Female | Prevalence | 625.1694491 | 450.1604369 | 800.1784613 |
| 1990 | Both | Prevalence | 1226.294709 | 1220.618261 | 1231.971157 |
| 1991 | Both | Prevalence | 1201.416294 | 1195.868425 | 1206.964164 |
| 1992 | Both | Prevalence | 1180.817676 | 1175.385413 | 1186.249938 |
| 1993 | Both | Prevalence | 1165.240185 | 1159.911062 | 1170.569309 |
| 1994 | Both | Prevalence | 1154.915967 | 1149.688005 | 1160.143928 |
| 1995 | Both | Prevalence | 1150.301475 | 1145.151646 | 1155.451304 |
| 1996 | Both | Prevalence | 1150.965105 | 1145.884845 | 1156.045365 |
| 1997 | Both | Prevalence | 1155.67326 | 1150.649618 | 1160.696902 |
| 1998 | Both | Prevalence | 1163.377933 | 1158.397155 | 1168.358711 |
| 1999 | Both | Prevalence | 1172.134948 | 1167.201971 | 1177.067926 |
| 2000 | Both | Prevalence | 1181.672833 | 1176.774232 | 1186.571434 |
| 2001 | Both | Prevalence | 1194.265977 | 1189.411605 | 1199.12035 |
| 2002 | Both | Prevalence | 1212.001322 | 1207.196532 | 1216.806112 |
| 2003 | Both | Prevalence | 1231.587673 | 1226.839434 | 1236.335912 |
| 2004 | Both | Prevalence | 1250.531924 | 1245.856574 | 1255.207273 |
| 2005 | Both | Prevalence | 1265.160853 | 1260.546923 | 1269.774783 |
| 2006 | Both | Prevalence | 1275.484726 | 1270.932894 | 1280.036557 |
| 2007 | Both | Prevalence | 1284.969344 | 1280.478893 | 1289.459795 |
| 2008 | Both | Prevalence | 1294.445345 | 1290.024511 | 1298.866179 |
| 2009 | Both | Prevalence | 1304.886431 | 1300.503546 | 1309.269316 |
| 2010 | Both | Prevalence | 1317.152329 | 1312.809078 | 1321.495579 |
| 2011 | Both | Prevalence | 1335.626359 | 1331.299144 | 1339.953575 |
| 2012 | Both | Prevalence | 1360.640092 | 1356.321216 | 1364.958969 |
| 2013 | Both | Prevalence | 1387.737791 | 1383.421132 | 1392.05445 |
| 2014 | Both | Prevalence | 1412.70866 | 1408.382403 | 1417.034917 |
| 2015 | Both | Prevalence | 1431.197433 | 1426.854083 | 1435.540783 |
| 2016 | Both | Prevalence | 1445.407227 | 1441.047065 | 1449.767389 |
| 2017 | Both | Prevalence | 1459.643668 | 1455.274849 | 1464.012487 |
| 2018 | Both | Prevalence | 1472.603165 | 1468.240861 | 1476.965469 |
| 2019 | Both | Prevalence | 1483.175367 | 1478.824027 | 1487.526707 |
| 2020 | Both | Prevalence | 1490.021302 | 1485.677354 | 1494.365251 |
| 2021 | Both | Prevalence | 1494.935131 | 1490.585469 | 1499.284793 |
| 2022 | Both | Prevalence | 1496.544772 | 1492.179659 | 1500.909884 |
| 2023 | Both | Prevalence | 1484.842949 | 1480.474241 | 1489.211657 |
| 2024 | Both | Prevalence | 1483.642579 | 1426.830497 | 1540.45466 |
| 2025 | Both | Prevalence | 1480.146157 | 1406.365832 | 1553.926483 |
| 2026 | Both | Prevalence | 1476.490697 | 1379.421332 | 1573.560062 |
| 2027 | Both | Prevalence | 1472.568731 | 1347.363644 | 1597.773818 |
| 2028 | Both | Prevalence | 1468.317046 | 1310.99514 | 1625.638952 |
| 2029 | Both | Prevalence | 1464.113935 | 1271.144307 | 1657.083563 |
| 2030 | Both | Prevalence | 1460.294184 | 1228.658121 | 1691.930247 |
| 2031 | Both | Prevalence | 1457.100469 | 1184.040243 | 1730.160696 |
| 2032 | Both | Prevalence | 1454.701858 | 1137.53039 | 1771.873327 |
| 2033 | Both | Prevalence | 1453.05149 | 1089.04971 | 1817.05327 |
| 2034 | Both | Prevalence | 1452.223704 | 1038.554976 | 1865.892433 |
| 2035 | Both | Prevalence | 1452.443631 | 986.2159178 | 1918.671345 |
