# Supplementary material for: Trends, decomposition, and projections of gout burden among middle-aged adults in China: GBD 2023
Source: Medicine (Baltimore). 2026 Oct 9;105(41):e51086. doi: 10.1097/MD.0000000000051086 (PMC13657502; doi:10.1097/MD.0000000000051086)
Supplement: Supplementary file 7 [file medi-105-e51086-s008.docx]

**Supplementary Table S7. Population attributable fractions of gout DALYs attributable to high body-mass index and kidney dysfunction among Chinese adults aged 40–59 years, by age group and sex, 1990 and 2023**

| **Risk factor** | **Age group** | **Sex** | **PAF in 1990, % (95% UI)** | **PAF in 2023, % (95% UI)** | **Absolute change, percentage points** | **Relative change, %** |
| --- | --- | --- | --- | --- | --- | --- |
| High body-mass index | 40-44 years | Male | 24.3 (17.9–32.2) | 34.1 (24.8–42.7) | 9.8 | 40.4 |
| High body-mass index | 40-44 years | Female | 21.7 (15.5–29.9) | 31.1 (22.5–40.0) | 9.4 | 43.4 |
| High body-mass index | 40-44 years | Both | 23.8 (17.5–31.8) | 33.5 (24.4–42.3) | 9.7 | 40.9 |
| High body-mass index | 45-49 years | Male | 20.2 (14.5–27.4) | 32.0 (23.7–40.9) | 11.8 | 58.2 |
| High body-mass index | 45-49 years | Female | 21.1 (14.8–29.0) | 32.5 (22.7–42.0) | 11.4 | 54.1 |
| High body-mass index | 45-49 years | Both | 20.4 (14.6–27.8) | 32.1 (23.4–41.0) | 11.7 | 57.4 |
| High body-mass index | 50-54 years | Male | 20.8 (15.1–27.9) | 31.0 (23.1–39.3) | 10.2 | 49 |
| High body-mass index | 50-54 years | Female | 23.1 (16.1–31.7) | 33.8 (24.1–43.2) | 10.7 | 46.3 |
| High body-mass index | 50-54 years | Both | 21.3 (15.4–28.7) | 31.6 (23.3–40.0) | 10.3 | 48.5 |
| High body-mass index | 55-59 years | Male | 21.1 (15.3–28.4) | 30.7 (22.7–38.7) | 9.5 | 45 |
| High body-mass index | 55-59 years | Female | 24.3 (17.3–32.5) | 33.8 (24.4–43.1) | 9.5 | 38.9 |
| High body-mass index | 55-59 years | Both | 21.9 (15.7–29.2) | 31.4 (23.0–39.7) | 9.5 | 43.6 |
| Kidney dysfunction | 40-44 years | Male | 3.1 (-0.1–10.9) | 2.5 (-0.1–8.6) | -0.6 | -19.5 |
| Kidney dysfunction | 40-44 years | Female | 4.1 (-0.1–14.0) | 2.9 (-0.1–9.9) | -1.3 | -30.6 |
| Kidney dysfunction | 40-44 years | Both | 3.3 (-0.1–11.5) | 2.6 (-0.1–8.8) | -0.7 | -22 |
| Kidney dysfunction | 45-49 years | Male | 4.7 (-0.1–15.2) | 3.6 (-0.1–12.2) | -1.1 | -22.4 |
| Kidney dysfunction | 45-49 years | Female | 6.0 (-0.2–19.0) | 4.0 (-0.1–13.1) | -2 | -33.5 |
| Kidney dysfunction | 45-49 years | Both | 4.9 (-0.1–15.9) | 3.7 (-0.1–12.4) | -1.2 | -25 |
| Kidney dysfunction | 50-54 years | Male | 7.0 (-0.2–22.7) | 5.4 (-0.1–18.0) | -1.6 | -22.9 |
| Kidney dysfunction | 50-54 years | Female | 8.7 (-0.2–27.3) | 5.8 (-0.1–19.1) | -2.9 | -33.7 |
| Kidney dysfunction | 50-54 years | Both | 7.3 (-0.2–23.6) | 5.5 (-0.1–18.2) | -1.9 | -25.5 |
| Kidney dysfunction | 55-59 years | Male | 10.0 (-0.2–31.3) | 7.8 (-0.2–25.2) | -2.2 | -21.7 |
| Kidney dysfunction | 55-59 years | Female | 12.2 (-0.3–37.2) | 8.4 (-0.2–26.6) | -3.9 | -31.6 |
| Kidney dysfunction | 55-59 years | Both | 10.5 (-0.3–32.6) | 8.0 (-0.2–25.5) | -2.5 | -24.2 |

Abbreviations: DALYs, disability-adjusted life-years; PAF, population attributable fraction; UI, uncertainty interval. PAF values were taken directly from the IHME GBD 2023 records (metric = Percent) and multiplied by 100. Absolute change was calculated as the 2023 PAF minus the 1990 PAF; relative change was calculated as (PAF2023 − PAF1990) / PAF1990 × 100%. Negative lower uncertainty bounds for kidney dysfunction were retained as reported in the source data.
